# Preclinical Evaluation of an Imidazole-Linked Heterocycle for Alzheimer’s Disease

**DOI:** 10.3390/pharmaceutics15102381

**Published:** 2023-09-25

**Authors:** Andrea Bagán, Sergio Rodriguez-Arévalo, Teresa Taboada-Jara, Christian Griñán-Ferré, Mercè Pallàs, Iria Brocos-Mosquera, Luis F. Callado, José A. Morales-García, Belén Pérez, Caridad Diaz, Rosario Fernández-Godino, Olga Genilloud, Milan Beljkas, Slavica Oljacic, Katarina Nikolic, Carmen Escolano

**Affiliations:** 1 Laboratory of Medicinal Chemistry (Associated Unit to CSIC), Department of Pharmacology, Toxicology and Medicinal Chemistry, Faculty of Pharmacy and Food Sciences, Institute of Biomedicine (IBUB), University of Barcelona, Av. Joan XXIII, 27-31, 08028 Barcelona, Spain; andreabaganp@gmail.com (A.B.); sergio6_6@hotmail.com (S.R.-A.); 2 Pharmacology Section, Toxicology and Medicinal Chemistry, Faculty of Pharmacy and Food Sciences, Institut de Neurociències, University of Barcelona, Av. Joan XXIII, 27-31, 08028 Barcelona, Spain; teretaboada93@gmail.com (T.T.-J.); christian.grinan@ub.edu (C.G.-F.); pallas@ub.edu (M.P.); 3 Centro de Investigación Biomédica en Red Enfermedades Neurodegenerativas (CiberNed), National Institute of Health Carlos III, 28029 Madrid, Spain; 4 Department of Pharmacology, University of the Basque Country, UPV/EHU, 48940 Leioa, Spain; iria.brocos@ehu.eus (I.B.-M.); lf.callado@ehu.eus (L.F.C.); 5 Centro de Investigación Biomédica en Red de Salud Mental, CIBERSAM, 28029 Madrid, Spain; 6 Biocruces Bizkaia Health Research Institute, 48903 Barakaldo, Spain; 7 Department of Cell Biology, School of Medicine, Complutense University (UCM), 28040 Madrid, Spain; jmoral06@ucm.es; 8 Department of Pharmacology, Therapeutic and Toxicology, Autonomous University of Barcelona, 08193 Cerdanyola, Spain; belen.perez@uab.cat; 9 Fundación MEDINA Centro de Excelencia en Investigación de Medicamentos Innovadores en Andalucía, Avda. del Conocimiento 34, 18016 Armilla, Spain; caridad.diaz@medinaandalucia.es (C.D.); rosario.fernandez@medinaandalucia.es (R.F.-G.); olga.genilloud@medinaandalucia.es (O.G.); 10 Department of Pharmaceutical Chemistry, Faculty of Pharmacy, University of Belgrade, Vojvode Stepe 450, 11221 Belgrade, Serbia; milan.beljkas@pharmacy.bg.ac.rs (M.B.); slavica.oljacic@pharmacy.bg.ac.rs (S.O.); katarina.nikolic@pharmacy.bg.ac.rs (K.N.)

**Keywords:** imidazoline I_2_ receptor ligand, Alzheimer’s disease, imidazoline-linked heterocycle, 2-(benzo[*b*]thiophen-2-yl)-1*H*-imidazole, 5XFAD, 3D-QSAR, neuroprotection

## Abstract

Humanity is facing a vast prevalence of neurodegenerative diseases, with Alzheimer’s disease (AD) being the most dominant, without efficacious drugs, and with only a few therapeutic targets identified. In this scenario, we aim to find molecular entities that modulate imidazoline I_2_ receptors (I_2_-IRs) that have been pointed out as relevant targets in AD. In this work, we explored structural modifications of well-established I_2_-IR ligands, giving access to derivatives with an imidazole-linked heterocycle as a common key feature. We report the synthesis, the affinity in human I_2_-IRs, the brain penetration capabilities, the in silico ADMET studies, and the three-dimensional quantitative structure-activity relationship (3D-QSAR) studies of this new bunch of I_2_-IR ligands. Selected compounds showed neuroprotective properties and beneficial effects in an in vitro model of Parkinson’s disease, rescued the human dopaminergic cell line SH-SY5Y from death after treatment with 6-hydroxydopamine, and showed crucial anti-inflammatory effects in a cellular model of neuroinflammation. After a preliminary pharmacokinetic study, we explored the action of our representative 2-(benzo[*b*]thiophen-2-yl)-1*H*-imidazole **LSL33** in a mouse model of AD (5xFAD). Oral administration of **LSL33** at 2 mg/Kg for 4 weeks ameliorated 5XFAD cognitive impairment and synaptic plasticity, as well as reduced neuroinflammation markers. In summary, this new I_2_-IR ligand that promoted beneficial effects in a well-established AD mouse model should be considered a promising therapeutic strategy for neurodegeneration.

## 1. Introduction

A longer life expectancy has become a worldwide challenge owing to the increased susceptibility to develop chronic and degenerative diseases that come with aging [1,2]. Alzheimer’s disease (AD) is an irreversible and incurable neurodegenerative disorder clinically characterized by progressive behavioral disturbances and memory loss [3]. The two major neuropathological AD markers that lead to synaptic failure are amyloid-β plaques and neurofibrillary tangles, consisting of hyperphosphorylated tau (p-Tau), that also trigger the inflammatory response [4]. Thus, targeting amyloid-β aggregation, p-Tau, and neuroinflammation has been proven to be the main disease-modifying strategy to treat AD so far. The progress made in target identification, drug discovery, and clinical trial methodology has increased our confidence in the development of new curative strategies. However, even though the drug development pipeline in 2022 dates back nearly 150 years [5], many potential curative strategies have failed, including the most promising Aβ-directed therapies [6,7].

So far, only symptomatic treatments, including the acetylcholinesterase inhibitors and the *N*-methyl-D-aspartate antagonists, are available for AD therapy. These drugs lack curative effects as they do not halt the progression of AD. They do, however, show modest symptomatic benefits for behaviour and cognition.

Currently, research endeavors are directed towards (1) identifying new molecular targets and the in-depth study of their physiological responses after their modulation, (2) detecting AD using artificial intelligence [8], or (3) paying attention to repurposing approved drugs to treat AD [9].

**Figure 1:**
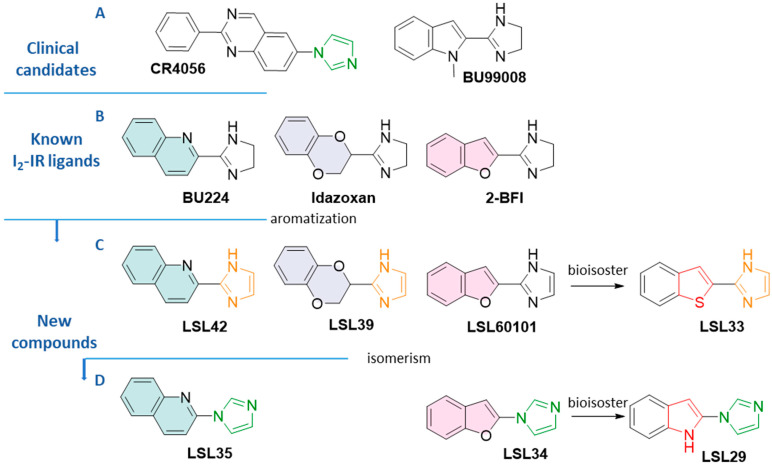
(**A**) Structure of clinical candidates CR4056 and [^11^C]BU99008. (**B**) Structure of standard I_2_-IR ligands BU224, idazoxan, and 2-BFI (**C**) Structure of compounds **LSL42**, **LSL39**, LSL60101, and **LSL33** (**D**) Structure of compounds **LSL35**, **LSL34**, **LSL29**, and **LSL33** The aromatization, isomerism, and bioisoster relationships are indicated.

In this context, we focused on relevant AD targets such as imidazoline I_2_ receptors (I_2_-IRs), which are expressed in both neurons and glial cells in the central nervous system (CNS) [10,11] and are found in a larger proportion in the brain of AD patients [12]. I_2_-IRs are orphan from the structural point of view, and their characterization relies on the affinity for radiolabeled ligands such as [^3^H]idazoxan and [^3^H]2-BFI [13]. The modulation of I_2_-IR by selective ligands [14], such as idazoxan, 2-BFI, or BU224 (Figure 1B), has functional responses in analgesia [15], inflammation, and severe human brain disorders [16], such as depression, gliomas [17], Huntington disease [18] and Parkinson disease (PD) [19]. Although these physiological events are well described, less is known about the upstream mechanism involved in the beneficial effects of the modulation of the I_2_-IRs.

Noteworthy, if CR4056, which is in a phase II multisite randomized placebo-controlled clinical trial in knee osteoarthritis patients, is eventually approved, we would find ourselves in front with an analgesic with a complete novel mechanism of action as well as the first-in-class drug based on I_2_-IRs pharmacology [20]. The I_2_-IR ligand ^11^C-BU99008 is a clinical candidate in phase I for Positron Emission Tomography (PET) diagnostics in patients suffering from AD (Figure 1A) [21]. 

In this scenario, our group provided several lines of evidence that demonstrate that the I_2_-IR’s modulation with original structurally selective ligands, from the families of (2-imidazolin-4-yl)phosphonates [22] and bicyclic α-iminophosphonates [23], improves behavioral and psychological symptoms of dementia, including fear-anxiety, depressive-like behavior, and memory decline, and ameliorates AD pathological features in well-established animal models of neurodegeneration and AD [24,25,26,27].

In a more conservative fashion, having considered structural patterns of known ligands, we explored modifications on the benzofuranyl-2-imidazole nucleus of compound LSL60101 (Figure 1C) [28], which was proposed by our group as a disease-modifying treatment in an AD mouse model and compared with the gold-standard anti-ADdonepezil [29]. 

Herein, we describe the synthesis and pharmacological characteristics of new I_2_-IR ligands based on the combination of the structure of LSL60101 and the standard ligands depicted in Figure 1. In line with the known pivotal role of nitrogenated heterocycles in drug discovery for Alzheimer’s disease [30], our final compounds possess a skeleton with an imidazole connected to a heterocycle inspired by the criteria of bioisosterism (Figure 1). The bioisosteric approach is a strategy used by medicinal chemists to address the design and development of a therapeutic agent with improved pharmacological properties and the bonus of possibly generating novel intellectual property [31].

Considering the localization of the I_2_-IRs in the CNS and our interest in proposing anti-AD agents, the ability of compounds to cross the blood-brain barrier (BBB) was an essential requirement. Therefore, we evaluated the Pe value in a parallel artificial membrane permeability assay (PAMPA), as well as the pharmacological profile of final compounds and selectivity through competition-binding studies against the selective radioligand of the I_2_-IR, [^3^H]2BFI. Moreover, I_2_-IRs are often described as non-adrenergic binding sites for imidazolines [32], so the selectivity versus the related target α_2_-adrenergic receptor (α_2_-AR) was evaluated through competition studies using the selective radioligand [^3^H]RX821002 (2-methoxyidazoxan). Complementarily, a three-dimensional quantitative structure-activity relationship (3D-QSAR) of this family was performed to delineate the pharmacophore as well as to have a starting point for future modifications in the structure, expecting improvement in the activities. The in silico ADME studies were carried out before undertaking in vitro assays.

In vitro models are laboratory-based experimental systems that allow us to study specific aspects of AD and PD in a controlled environment outside of living organisms. These models provide valuable tools for testing potential therapies and identifying novel targets for drug development. In this work, we employed two well-known preclinical models to study neuronal degeneration, specifically targeting AD and PD. Additionally, considering the significant influence exerted by neuroinflammation in the pathogenesis of these disorders, we conducted an analysis to assess the potential involvement of I_2_-IR ligands in glial cell lines and isolated microglial and astroglial cultures obtained from the cerebral cortex of mice. Derived from the outcomes acquired, we selected 2-(benzo[*b*]thiophen-2-yl)-1*H*-imidazole, named **LSL33**, for the in vivo experiments. The imidazole has been recognized as a preferred structure in active compounds [33]. The benzo[*b*]thiophene nucleus has demonstrated interest in a plethora of therapeutic indications [34], and more conveniently, benzo[*b*]thiophene derivatives have been used as potential diagnostic agents in AD [35,36] and investigated in PD therapy [37,38].

Next, a preliminary pharmacokinetic (PK) profile was performed before carrying out in vivo experiments in the familial AD murine model 5xFAD. The latter represents a well-established transgenic model that, as early as 2 months, develops early and aggressive hallmarks of amyloid burden and cognitive impairment [39]. Additional AD pathologies exhibited by the 5XFAD model are synaptic dysfunction [40] and an increase in neuroinflammation [41]. The results of the in vivo experiments situate **LSL33** as a promising representative anti-AD compound that belongs to the imidazole-linked heterocycle family described in this paper.

## 2. Materials and Methods

### 2.1. Chemistry

For detailed experimental conditions, see Supplementary Materials.

#### 2.1.1. Synthesis of **LSL42**, **LSL39** and **LSL33** [42]

General procedure for the synthesis of 2-carbimididate hydrochloride derivatives **2a**, **2b**, and **2c**.

The 2-cyanobenzofuran derivatives **1a**, **1b**, or **1c** (1 equiv) were dissolved in ethereal 2 M HCl (0.25 mmol/mL) and methanol (5 mmol/mL). The resulting mixture was kept at 4 °C for 48 h. The resulting solid was filtered, washed with cold diethyl ether, and dried in order to obtain the carbimidate hydrochloride **2a**, **2b**, or **2c**. Compounds were used without further purification in the next synthetic step.

**Methyl quinoline-2-carbimidate hydrochloride (2a)** Following the general procedure, quinoline-2-carbonitrile **1a** (500 mg, 3.25 mmol), ethereal 2 M HCl (13 mL), and methanol (0.65 mL) gave **2a** (555 mg, 77%) as a white solid.

**Methyl 2,3-dihydrobenzo[1,4]dioxine-2-carbimidate hydrochloride (2b)**. Following the general procedure, 2,3-dihydro-1,4-benzodioxine-2-carbonitrile **1b** (500 mg, 3.10 mmol), ethereal 2 M HCl (12.5 mL), and methanol (0.62 mL) gave **2b** (668 mg, 94%) as a white solid.

**Methyl benzothiophene-2-carbimidate hydrochloride (2c)** Following the general procedure, benzothiophene-2-carbonitrile **1c** (500 mg, 3.14 mmol), ethereal 2 M HCl (12.6 mL), and methanol (0.63 mL) gave **2c** (608 mg, 85%) as a white solid.

General procedure for the synthesis of N-(2,2-dimethoxyethyl)-2-carboximidamide derivatives **3a**, **3b**, and **3c**.

A solution of 2,2-dimethoxyethylamine (1.1 equiv) and 2-carbimidate hydrochloride derivatives **2a**, **2b**, or **2c** (1 equiv) in methanol (0.47 mmol/mL) was stirred at 60 °C for 16 h. The mixture was evaporated to dryness and used directly in the next step without further purification.

***N*-(2,2-Dimethoxyethyl)quinoline-2-carboximidamide (3a)**. Following the general procedure, **2a** (500 mg, 2.25 mmol), 2,2-dimethoxyethylamine (0.27 mL, 2.48 mmol), and methanol (5.4 mL) gave **3a** (583 mg, quantitative yield) as a beige solid.

***N*-(2,2-Dimethoxyethyl)-2,3-dihydrobenzo[1,4]dioxine-2-carboximidamide (3b)**. Following the general procedure, **2b** (650 mg, 2.84 mmol), 2,2-dimethoxyethylamine (0.34 mL, 3.12 mmol), and methanol (6.0 mL) gave **3b** (755 mg, quantitative yield) as a beige solid.

***N*-(2,2-Dimethoxyethyl)benzothiophene-2-carboximidamide (3c)**. Following the general procedure, **2c** (590 mg, 2.60 mmol), 2,2-dimethoxyethylamine (0.31 mL, 2.86 mmol), and methanol (5.5 mL) gave **3c** (687 mg, quantitative yield) as a beige solid.

General procedure for the synthesis of 1H-imidazole hydrochloride derivatives **LSL42**, **LSL39**, and **LSL33**.

The *N*-(2,2-dimethoxyethyl)-2-carboximidamide **3a**, **3b**, or **3c** (1 equiv) was treated with 2M HCl (0.1 mmol/mL), and the resulting mixture was stirred at 60 °C for 16 h. After cooling, the solution was washed with DCM. The aqueous layer was basified with 5 M NaOH, and the free base was extracted with EtOAc. The combined organic phases were washed with brine, dried over Na_2_SO_4_, and evaporated to give a residue that was dissolved in diethyl ether/ethanol (5:1). Ethereal 2 M HCl (1.5 mmmol/mL) was added, and the precipitated salt was collected by filtration to give the desired product.

**2-(Quinoline-2-yl)-1H-imidazole hydrochloride (LSL42).**

Following the general procedure, **3a** (550 mg, 2.12 mmol), 2 M HCl (21.2 mL), and ethereal 2 M HCl (1.4 mL) gave **LSL42** (284 mg, 58%) as a white solid. IR (ATR) 3398, 2996, 2608, 1618, 1557, 1404, 1351, 1289, 1205, 1019, 803, 798, 721 cm^−1^. ^1^H-NMR (400 MHz, CD_3_OD) δ 7.75 (t, *J* = 8.0 Hz, 1H), 7.78 (s, 2H), 7.91 (t, *J* = 8.5 Hz, 1H), 8.06 (d, *J* = 8.0 Hz, 1H), 8.17 (d, *J* = 8.5 Hz, 1H), 8.23 (d, *J* = 9.0 Hz, 1H), and 8.63 (d, *J* = 8.5 Hz, 1H). HRMS C_12_H_10_N_3_ [M + H]^+^ 196.0869; found, 196.0873.

**2-(2,3-Dihydrobenzo[1,4]dioxin-2-yl)-1H-imidazole hydrochloride (LSL39)**.

Following the general procedure, **3b** (700 mg, 2.63 mmol), 2M HCl (26.3 mL), and ethereal 2 M HCl (1.8 mL) gave **LSL39** (401 mg, 64%) as a white solid. IR (ATR) 3365, 2877, 2673, 1595, 1490, 1262, 1074, 1041, 875, 751 cm^−1^. ^1^H-NMR (400 MHz, DMSO-*d*_6_) δ 4.64 (d, *J* = 5.0 Hz, 2H), 5.85 (t, *J* = 4.0 Hz, 1H), 6.92–7.04 (m, 5H), 7.74 (s, 2H). ^13^C-NMR (100.6 MHz, DMSO-*d*_6_) δ 64.4, 67.0, 117.4, 117.6, 120.2 (2C), 122.1, 122.4, 141.4, 141.6, 142.5. HRMS C_11_H_11_N_2_O_2_ [M + H]^+^ 203.0815; found, 203.0816.

**2-(Benzo[b]thiophene-2-yl)-1H-imidazole hydrochloride (LSL33).**

Following the general procedure, **3c** (680 mg, 2.57 mmol), 2 M HCl (25.7 mL), and ethereal 2 M HCl (1.7 mL) gave **LSL33** (492 mg, 81%) as a white solid. IR (ATR) 3395, 3172, 3146, 2562, 1623, 1455, 1445, 1140, 1094, 860, 842, 736 cm^−1^. ^1^H-NMR (400 MHz, DMSO-*d*_6_) δ 7.49–7.55 (m, 2H), 7.77 (s, 2H), 8.02 (d, *J* = 8.5 Hz, 1H), 8.15 (d, *J* = 8.5 Hz, 1H), 8.38 (s, 1H). ^13^C NMR (100.6 MHz, DMSO-*d*_6_) δ 120.8 (2C), 123.0, 124.9, 125.1, 125.7, 126.9, 127.3, 138.5, 138.6, 139.9. HRMS C_11_H_9_N_2_O [M + H]^+^ 201.0481; found, 201.0482. Purity: 99% (t_R_ = 3.27 min).

#### 2.1.2. Synthesis of **LSL35**, **LSL34** and **LSL29**

**1-(Quinoline-2-yl)-1H-imidazole hydrochloride (LSL35) [43]** **.**

To a solution of 2-bromoquinoline (104 mg, 0.5 mmol) in DMSO (2 mL), imidazole (17 mg, 0.25 mmol), HOBt (3.4 mg, 0.025 mmol), KOtBu (42.1 mg, 0.375 mmol), and CuI (2.5 mg, 0.013 mmol) were added. The reaction mixture was stirred under reflux for 24 h. After completion of the reaction, the resulting residue was filtered over Celite^®^ and washed with EtOAc. The resulting solution was washed with brine, dried over Na_2_SO_4_, and concentrated in vacuo. Then, the crude product was purified by flash chromatography (DCM 100%). The product was dissolved in DCM, ethereal 2M HCl (0.2 mL) was added, and the precipitated salt was collected by filtration to give **LSL35** (55 mg, 48%). IR (ATR) 3426, 3096, 3010, 2638, 1588, 1541, 1507, 1434, 1355, 1328, 1225, 989, 903, 821 cm^−1^. ^1^H-NMR (400 MHz, DMSO-*d*_6_): δ 7.74 (t, *J* = 8.0 Hz, 1H), 7.88 (s, 1H), 7.92 (t, *J* = 7.0 Hz, 1H), 8.07 (d, *J* = 8.5 Hz, 1H), 8.14 (d, *J* = 7.5 Hz, 1H), 8.21 (d, *J* = 9.0 Hz, 1H), 8.58 (s, 1H), 8.79 (d, *J* = 9.0 Hz, 1H), 9.98 (s, 1H). HRMS C_12_H_10_N_3_ [M + H]^+^ 196.0869; found, 196.0871. Purity: 100% (t_R_ = 3.34 min).

**1-(Benzofuran-2-yl)-1H-imidazole hydrochloride (LSL34) [44]**.

To a solution of 2-bromobenzofurane (197 mg, 1 mmol) in ACN (5 mL), imidazole (102 mg, 1.5 mmol), Cs_2_CO_3_ (651 mg, 2.0 mmol), and CuI (38 mg, 0.2 mmol) were added. The reaction mixture was stirred under reflux for 24 h. After completion of the reaction, the resulting residue was filtered over Celite^®^, washed with EtOAc, and concentrated in vacuo. Then, the crude product was purified by flash chromatography (DCM 100%). The product was dissolved in DCM, ethereal 2M HCl (0.7 mL) was added, and the precipitated salt was collected by filtration to give **LSL34** (121 mg, 55%). IR (ATR) 3385, 3075, 2883, 1905, 1628, 1579, 1536, 1455, 1389, 1198, 1056, 990, 801 cm^−1^. ^1^H-NMR (400 MHz, DMSO-*d*_6_): δ 7.04 (s, 1H), 7.19 (s, 1H), 7.29–7.37 (m, 2H), 7.62–7.65 (m, 1H), 7.68 (dd, *J* = 7.5, 1.5 Hz, 1H), 7.83 (s, 1H), 8.35 (s, 1H). HRMS C_11_H_9_N_2_O [M + H]^+^ 185.0711; found, 185.0711. Purity: 100% (t_R_ = 3.70 min).

**2-Imidazol-1-yl-1H-indole hydrochloride (LSL29)** [45].

Iodine (317 mg, 1.25 mmol) was added to a mixture of indole (59 mg, 0.5 mmol), imidazole (272 mg, 4 mmol), and a saturated aqueous ammonium formate solution (0.3 mL) in 1,4-dioxane (0.3 mL). The reaction mixture was stirred at room temperature for 24 h. Then, a saturated aqueous Na_2_S_2_O_3_ solution (1 mL) was added. The reaction mixture was extracted with EtOAc. The combined organic layer was washed with brine and dried over anhydrous Na_2_SO_4_, filtered, and concentrated in vacuo. The crude product was purified by flash chromatography on silica gel (DCM 100%). The product was dissolved in DCM, and an ethereal 2 M HCl (0.4 mL) was added. The precipitated salt was collected by filtration to give **LSL29** (35 mg, 32%). IR (ATR): 3105, 2688, 2617, 1579, 1564, 1391, 1328, 1236, 1068, 898, 824, 779 cm^−1^. ^1^H-NMR (400 MHz, DMSO-*d*_6_): δ 6.89 (d, *J* = 5.0 Hz, 1H), 7.12 (t, *J* = 7.0 Hz, 1H), 7.23 (t, *J* = 7.0 Hz, 1H), 7.47 (d, *J* = 8.5 Hz, 1H), 7.62 (d, *J* = 7.5 Hz, 1H), 7.84 (s, 1H), 8.26 (s, 1H), 9.59 (s, 1H), and 12.52 (br s, 1H). HRMS C_11_H_10_N_3_ [M + H]^+^ 184.0869; found, 184.0870. Purity: 96% (t_R_ = 3.37 min).

### 2.2. Binding Studies

#### 2.2.1. Preparation of Cellular Membranes

For detailed experimental conditions, see Supplementary Materials.

#### 2.2.2. Competition Binding Assays

For detailed experimental conditions see Supplementary Materials.

### 2.3. 3D-QSAR Study—Data Set Preparation

The study was performed on a data set of 31 compounds, including 6 accessed ligands, 6 known I_2_-IR ligands, and 19 ligands previously described by our group (10 bicyclic α-iminophosphonates and 9 benzofuranyl-2-imidazole derivatives [28]. From the structural chemistry point of view, the studied compounds are diverse and can be divided into two clusters, one containing imidazole and imidazoline derivatives and the other consisting of bicyclic α-iminophosphonates (Figure S1).

The initial molecular data set was divided into a training set for model building and a test set for external validation of the model. The 3D-QSAR (I_2_-IR) model was built with 31 compounds, 22 ligands in the training set, and 9 ligands in the test set, whereas the 3D-QSAR (α_2_-AR) model was built with 20 compounds in the training set and 9 compounds in the test set (Tables S1 and S2). The compounds in the test set were selected based on the Principal Component Analysis (PCA) plot, taking into account that the entire range of p*K*_i_ values is homogeneously distributed in both the training and test sets. Experimentally determined p*K*_i_ (I_2_-IR) and p*K*_i_ (α_2_-AR) were used as dependent variables in 3D-QSAR modeling and consistently ranged from 4.020 to 8.560, and 2.650 to 7.920 for 3D-QSAR (I_2_-IR) and 3D-QSAR (α_2_-AR), respectively.

The dominant forms of the studied ligands at a physiological pH of 7.4 were determined using the program Marvin Sketch 5.5.1.0 [46]. In the first step of geometry optimization, the semi-empirical/PM3 (Parameterized Model Revision 3) method [47,48], followed by the ab initio Hartree-Fock/3-21G method [49] was applied using the Gaussian 09 software [50] included in the ChemBio3D Ultra 13 program [51].

The calculation of alignment-independent three-dimensional molecular descriptors (GRIND) and the building of 3D-QSAR models were performed by using the Pentacle program [52]. The computation of descriptors is based on Molecular Interaction Fields (MIF), using four different probes: the O probe (hydrogen bond acceptor groups), the N1 probe (hydrogen bond donor groups), the TIP probe (the shape of the molecule), and the DRY probe (hydrophobic interactions). The algorithm ALMOND was used to select the most important MIFs that have favourable interaction positions between ligand and probe. In the last step, the CLACC (Consistently Large Auto and Cross Correlation) algorithm with a smoothing window of 0.8 Å was applied to display node-node energies, between the same or a different probe, into auto- and cross-correlograms [53]. Fractional factorial design (FFD) was used to select the most important variables, and Partial Least Square (PLS) regression was used to create the final 3D-QSAR models.

### 2.4. In Vitro Model of Alzheimer’s and Parkinsons’ Disease

In the study of neurotoxicity associated with AD, the HT22 mouse hippocampal neuronal cell line has been recognized as an effective cell model. HT22 cells were purchased from Merck-Sigma-Aldrich (SCC12, Madrid, Spain) and cultured in DMEM-F12 (Gibco, Fisher Scientific, Madrid, Spain) supplemented with foetal bovine serum (FBS, 10%) and 1% penicillin/streptomycin under standard conditions (37 °C, 5% CO_2_). The human dopaminergic cell line SH-SY5Y, in turn, has been widely used as an in vitro model to study the pathogenesis of PD associated with dopaminergic degeneration. Cells, acquired from the American Type Culture Collection (ATCC, Rockville, MD, USA, Ref.CRL-2266), were grown in RPMI medium (Gibco) supplemented with glutamine (2 mM), 10% FBS, and 1% penicillin/streptomycin under standard conditions. Semiconfluent cells (90%) were seeded on 96-well plates at 3 × 10^4^ per well and incubated for 24 h before treatment.

A large number of scientific studies have demonstrated that people with Alzheimer’s disease and mild cognitive impairment tend to have higher levels of proinflammatory factors [54]. PD has been described in the same way [55]. Therefore, we based our in vitro model of neuroinflammation on the use of two established glial cell lines, C6 and BV2, as well as primary glial cultures derived from the mouse cerebral cortex. C6 astroglia-like cell lines derived from rat brain (ATCC; CCL-107) and BV2 microglial cell lines derived from mice (Merk-Sigma Aldrich, Madrid, Spain) were cultured in DMEM with 10% FBS and 1% penicillin/streptomycin in a humidified atmosphere under standard conditions. When attaining semiconfluence, glial cells were seeded on 96-well plates at 3 × 10^4^/well and incubated for 24 h before treatment. Primary astrocytes and microglia were obtained and cultured following a previously described protocol [56]. In brief, the cerebral cortex was dissected, dissociated, and incubated with 0.25% trypsin and EDTA at 37 °C for 1 h. The tissue was then centrifuged, and the resulting pellet was washed with Hanks’ balanced salt solution (Invitrogen). The cells were plated on non-coated flasks and incubated under standard conditions for a minimum of 7 days. Subsequently, each type of glial cell was isolated to obtain pure cultures. To achieve this, the glial cells in the culture flasks were agitated on an orbital shaker for 4 h at 240 rpm and 37 °C. The supernatant was collected and centrifuged, and the resulting cellular pellet, containing the microglial cells, was resuspended in complete medium (HAMS/DMEM (1:1) containing 10% FBS) and seeded on uncoated 96-well plates. The cells were allowed to adhere for 2 h, after which the medium was removed to eliminate nonadherent oligodendrocytes. Fresh medium supplemented with 10 ng/mL of GM-CSF was added. The remaining astroglial cells adhered to the flasks were trypsinized, collected, centrifuged, and plated onto 96-well plates with complete medium. Immunofluorescence analysis using antibodies specific to Iba-1 (a microglial marker) and GFAP (an astrocyte marker) demonstrated that the cultures obtained through this procedure were more than 98% pure.

#### 2.4.1. Treatments

Neuronal cultures, HT-22 and SH-SY5Y, seeded on 96-well plates were treated with **LSL33**, **LSL34**, and LSL60101 for 24 h, and viability was assessed using the MTT assay. None of the compounds tested were cytotoxic at those doses. Then, cultures were treated for 1h with **LSL33** at 2 μM and **LSL34** and **LSL60101** at 1 μM. This dose was chosen based on previous studies. Subsequently, HT-22 cultures were exposed to glutamate (10 mM, Merck-Sigma), while SH-SY5Y cultures received 6-hydroxydopamine (6-OHDA, 35 μM, Merck-Sigma). The cells were then incubated for a minimum of 16 h. Finally, the cellular viability and nitrite production of the cultures were assessed.

To determine the inflammatory state, glial cells subcultured in 96-well plates were exposed to lipopolysaccharide (LPS, 10 μg/mL, Sigma). Some of the cultures were previously treated with I_2_-IR ligands for 1 h. After 24 h in culture, nitrite production was evaluated.

#### 2.4.2. Cell Viability Assay

The assessment of cell viability was conducted using the 3-(4,5-dimethylthiazol-2-yl)-5-(3-carboxymethoxyphenyl)-2-(4-sulfophenyl)-2H-tetrazolium (MTT) assay, following the instructions provided by the manufacturer (Roche Diagnostic, Darmstadt, Germany). Briefly, after exposure to glutamate or 6-OHDA, the cell culture medium was replaced with 100 μL of fresh culture medium containing MTT solution (0.5 mg/mL) and incubated for 1 h. The medium was then removed, and the resulting formazan crystals were dissolved in dimethylsulfoxide (DMSO, Merck-Sigma). The absorbance of the solution was measured at 595–650 nm using a spectrophotometer. The experiments were performed at least three times, and the results were expressed as a percentage of the control.

#### 2.4.3. Nitrite Measurement

Following the designated treatments, 100 μL of the cell culture supernatant was mixed with an equal volume of Griess reagent (consisting of 1% sulfanylamide and 0.1% naphthyl ethylene diamine in 5% phosphoric acid, sourced from Sigma-Aldrich, Madrid, Spain) at room temperature for 15 min. To determine the nitrite concentrations, a standard solution of sodium nitrite was employed. The absorbance of the mixture was measured at 492/540 nm using a microplate reader from Thermofisher (Madrid, Spain). The experiments were repeated a minimum of three times.

#### 2.4.4. Statistical Analysis

The results presented in the Section 3.5 (In vitro models of neurodegenerative diseases)are expressed as the mean ± standard deviation (SD) of triplicate determinations. The experiments were repeated at least three times, and consistent outcomes were obtained. Data analysis was initially performed using one-way analysis of variance (ANOVA). Subsequently, post hoc statistical analyses were conducted using the Tukey test, with a significance level set at *p* < 0.05. The SPSS statistical software package, version 20.0, for Windows (Chicago, IL, USA), was utilized for these analyses.

### 2.5. In Vivo Pharmacokinetics of **LSL33**

#### 2.5.1. Animals for PK Studies

For detailed experimental conditions, see Supplementary Materials.

#### 2.5.2. Method Validation for Quantitation of **LSL33** in Mouse Plasma and in Mouse Brain

The methods and data related to this section are included as Supplementary Materials.

### 2.6. In Vivo Experiments in Mice

Four-month-old female and male Wt and 5XFAD mice (*n*  =  32) were used to carry out cognitive and molecular analyses. The animals were randomly allocated to four experimental groups: the Wt Control group (Wt Control) (*n*  =  8) and the 5XFAD Control group (5XFAD Control) (*n* = 8), which were administered vehicle (2-hydroxypropyl)-β-cyclodextrin 1.8% in drinking water, and the Wt **LSL33** and 5XFAD **LSL33**, which were treated with the I_2_-IR ligand **LSL33** (2 mg/Kg) (*n*  =  8). The animals had free access to food and water and were kept under standard temperature conditions (22  ±  2 °C) and 12 h/12 h light/dark cycles (300 lx/0 lx). **LSL33** (2 mg/kg/day) was diluted in 1.8% (2-hydroxypropyl)-β-cyclodextrin and administered through drinking water. After 4 weeks of treatment, cognitive tests, including short- and long-term memory and OLT, were performed to study the effects of treatment on learning and memory. Weight and water consumption were controlled each week, and the **LSL33** concentration was adjusted accordingly to reach the optimal dose until euthanasia.

#### 2.6.1. Cognitive Tests

##### Novel Object Recognition Test (NORT)

Short- and long-term recognition memories involving cortical areas and the hippocampus were evaluated by NORT. The experimental apparatus used for this test was a 90-degree, two-arm, 25-cm-long, 20-cm-high maze of black polyvinyl chloride. The light intensity in the middle of the field was 30 lux. First, mice were individually habituated to the apparatus for 10 min per day for 3 days. On day 4, the animals were allowed to freely explore two identical objects (A and A or B and B) placed at the end of each arm for a 10 min acquisition trial (first trial-familiarization). Then, a 10-min retention trial (second trial) was carried out 2 h (short-term memory) or 24 h (long-term memory) later. During the short-term memory retention test, the time that the animal spent exploring the new object (TN) and the old object (TO) was recorded. Twenty-four hours after the acquisition trial, the mice were tested again with a new object and an object identical to the new one in the previous trial (A and C, or B and C). TN and TO were measured from the video recordings from each trial session. A discrimination index (DI) was defined as (TN − TO)/(TN + TO). The maze, the surface, and the objects were cleaned with 70% ethanol between the animals’ trials to eliminate olfactory cues.

##### Object Location Test (OLT)

The OLT evaluated the spontaneous tendency of rodents to spend more time exploring a novel object location than a familiar object location and recognizing when an object has been relocated. OLT was performed using a white plywood apparatus (50 × 50 × 25 cm), in which three walls were white and one was black. On the first day, animals were just habituated to the empty open-field arena for 10 min. On the second day, two objects were placed in front of the black wall, equidistant from each other and the wall. The objects were 10 cm high and identical. The animals were placed in the open-field arena and allowed to explore both objects and their surroundings for 10 min. Afterward, animals were returned to their home cages, and the OLT apparatus was cleaned with 70% ethanol. On the third day, one object was moved in front of the opposite white wall to test spatial memory. Trials were recorded using a camera mounted above the open field area, and the total exploration time was determined by scoring TN and TO. DI was calculated, defined as (TN−TO)/(TN+TO).

### 2.7. Molecular Experiments

#### 2.7.1. Brain Processing

Three days after cognitive tests, mice were euthanized for protein extraction and RNA and DNA isolation. After euthanasia, the brains were immediately removed from the skulls, and the hippocampi were dissected, frozen, and maintained at −80 °C.

#### 2.7.2. Protein Levels Determination by Western Blotting (WB)

For western blotting (WB), aliquots of 20 μg of hippocampal protein were used. Protein samples from mice were separated by sodium dodecyl sulfate-polyacrylamide gel electrophoresis (SDS-PAGE) (8–12%) and transferred onto polyvinylidene difluoride (PVDF) membranes (Millipore, Burlington, MA, USA). Afterwards, the membranes were blocked in 5% nonfat milk in 0.1% Tris-buffered saline with Tween 20 (TBS-T) for 1 h at room temperature before being incubated overnight at 4 °C with the primary antibodies listed in Table S18.

The membranes were washed and incubated with secondary antibodies for 1 h at room temperature. Immunoreactive proteins were viewed with a chemiluminescence-based detection kit following the manufacturer’s protocol (ECL Kit; Millipore), and digital images were acquired using a ChemiDoc XRS+ System (Bio-Rad, Hercules, CA, USA). Semiquantitative analyses were carried out using Image Lab software (Bio-Rad, 6.1. version), and the results are expressed in arbitrary units (AU), with the control protein levels set at 100%. Protein loading was routinely monitored by immunodetection of glyceraldehyde-3-phosphate dehydrogenase (GAPDH).

#### 2.7.3. RNA Extraction and Gene Expression Determination

Total RNA isolation was carried out using TRIzol^®^ reagent according to the manufacturer’s instructions. The yield, purity, and quality of RNA were determined spectrophotometrically with a NanoDrop™ ND-1000 apparatus (Thermo Scientific, Waltham, MA, USA) and an Agilent 2100B Bioanalyzer (Agilent Technologies, Santa Clara, CA, USA). RNA samples with 260/280 ratios and RNA integrity numbers (RINs) higher than 1.9 and 7.5, respectively, were selected. A reverse transcription-polymerase chain reaction (RT-PCR) was performed. Briefly, 2 μg of messenger RNA (mRNA) was reverse-transcribed using a high-capacity cDNA reverse transcription kit (Applied Biosystems, Foster City, CA, USA).

SYBR^®^ Green real-time PCR was performed on a StepOnePlus Detection System (Applied Biosystems) with SYBR^®^ Green PCR master mix (Applied Biosystems). Each reaction mixture contained 6.75 μL of complementary DNA (cDNA) (with a concentration of 2 μg), 0.75 μL of each primer (with a concentration of 100 nM), and 6.75 μL of SYBR^®^ Green PCR master mix (2×).

The data were analyzed utilizing the comparative cycle threshold (Ct) (ΔΔCt) method, in which the levels of a housekeeping gene are used to normalize differences in sample loading and preparation. Normalization of expression levels was performed with *β-actin* for SYBR^®^ Green-based real-time PCR. The primer sequences used in this study are presented in Table S19. Each sample was analyzed in duplicate, and the results represent the n-fold differences in the transcript levels among different groups.

## 3. Results and Discussion

### 3.1. Chemistry

Recently, we described the synthetic sequence to access compound LSL60101, which is the aromatic counterpart of 2BFI, where imidazoline has been replaced by imidazole [28]. Encouraged by the reduced cognitive impairment and the improved memory test results in 5xFAD after being treated with LSL60101 [29], we prepared the compounds **LSL42** and **LSL39**, which are the aromatic partners of BU224 and idazoxan, respectively (Figure 1C).

**Scheme 1:**
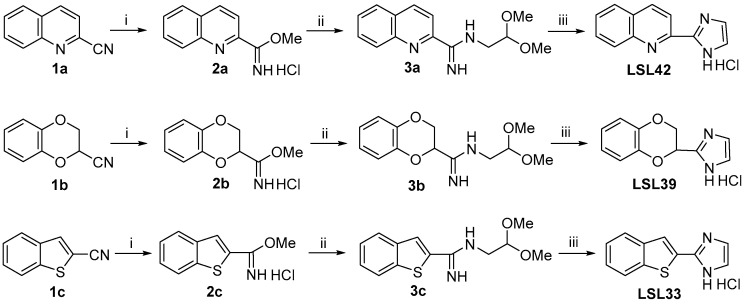
Condition reactions and yields for the synthesis of **LSL42**, **LSL39**, and **LSL23** (i) Et_2_O/HCl 2 M (0.25 mmol/mL), MeOH, 4 °C, 48 h, **2a**, 77%; **2b**, 94%; **2c**, 85% yield, respectively. (ii) 2,2-Dimethoxyethylamine (1.1 equiv.), MeOH, 60 °C, 16 h, quantitative for **3a**, **3b**, and **3c**. (iii) 2 M HCl (0.1 mmol/mL), 60 °C, 16 h, **LSL42**, 58%; **LSL39**, 64%; **LSL33**, 81% yield, respectively.

The preparation of these compounds, **LSL42** and **LSL39**, was undertaken starting from the corresponding commercially available quinone-2-carbonitrile, **1a**, and 2,3-dihydro-1,4-benzodioxine-2-carbonitrile, **1b**, based on a previously described procedure [28]. The carbonitrile derivatives were efficiently transformed after being treated with ethereal 2 M HCl in the quinoline-2-carbimidate **2a** and the 2,3-dihydrobenzo[1,4]dioxine-2-carbimidate **2b** in a 77% and 94% yield, respectively. To construct the imidazole moiety, the reaction with 2,2-dimethoxyethylamine was undertaken to give **3a** and **3b** in a quantitative yield. These were treated with aqueous hydrochloric acid to complete the attack of the nitrogen atom on the ketal electrophilic carbon, affording the compounds **LSL42** and **LSL39** a 58 and 64% yield, respectively (Scheme 1). The potential of LSL60101 led us to propose the bioisoster **LSL33**, where the benzofuran aromatic nucleus is substituted by benzo[*b*]thiophene. To access **LSL33**, we followed an analogous synthetic sequence as described above. The benzothiophene-2-carbonitrile **1c** was transformed to an 85% yield in the carbimidate **2c**. The reaction of **2c** with 2,2-dimethoxyethylamine led quantitatively to compound **3c,** which, after treatment with a solution of 2 M hydrochloric acid, gave **LSL33** in an 81% yield (Scheme 1).

**Scheme 2:**
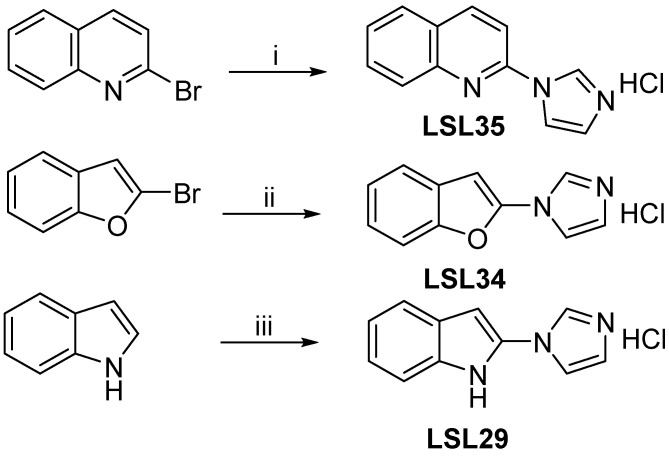
Condition reactions and yields for the synthesis of **LSL35**, **LSL34**, and **LSL29** (i) 2-Bromoquinoline (0.5 mmol), imidazole (0.25 mmol), HOBt (0.025 mmol), CuI (0.013 mmol), KOtBu (0.38 mmol), DMSO, reflux, 24 h, **LSL35**, 48% yield. (ii) 2-Bromobenzofurane (1 mmol), imidazole (1.5 mmol), CuI (0.2 mmol), Cs_2_CO_3_ (2 mmol), acetonitrile, reflux, 24 h, **LSL34**, 55% yield. (iii) Indole (0.5 mmol), imidazole (4 mmol), HCO_2_NH_4_, I_2_ (1.25 mmol), dioxane, rt, 24 h, **LSL29**, 32% yield.

To access the compounds in Figure 1D, we followed previously reported procedures. The practical and inexpensive ligand benzotriazole (10% mmol) promoted the copper- catalyzed *N*-arylation of imidazole with 2-bromoquinoline in DMSO by using potassium *tert*-butoxide as a base, leading to **LSL35** in a 48% yield [43]. The compound features a quinoline nucleus, a heterocycle present in BU224, and the *N*1-linked imidazole moiety that occurs in the clinical candidate CR40546 (Scheme 2).

Continuing with the idea of evaluating compounds inspired by the CR4056 structure, we prepared compounds **LSL34**, with the *N*1-imidazole linked to a benzofuran, and **LSL29,** with the *N*1-imidazole linked to an indole. Note that benzofuran and indole have been identified as promising scaffolds for drug development in AD [57]. The *N*-arylation (2-bromobenzofuran) of imidazole in the presence of copper salt (0.2 equivalents) and Cs_2_CO_3_ (2 equivalents) using acetonitrile as solvent (no other ligand) provided after 24 h of reflux the expected *N*-arylated indol-based **LSL34** with a 55% yield [44]. After treatment of indol with an excess of both imidazole and iodine in the presence of ammonium formate in 1,4-dioxane, compound **LSL29** was produced in 32% yield using a regioselective metal-free method for direct C-N bond formation from indoles and imidazole [45].

All reported compounds were accessed efficiently and were fully characterized (see Section 2 and Supplementary Materials).

### 3.2. In Silico ADMET Analysis of Physicochemical and Pharmacokinetic Parameters

To reduce investments and attrition rates and be able to optimize lead compounds, the drug discovery pipeline relies on in silico predictions [58]. Thus, the physicochemical properties were evaluated using the online program Swiss ADME (Table 1) [59]. Final compounds satisfy the rules proposed by Lipinski (Rule of Five), which is indicative of their drug-likeness and potential chance of oral bioavailability. Moreover, water solubility, lipophilicity, and topological polar surface area were within the proposed limits, indicating satisfactory physicochemical properties of the studied compounds.

In silico absorption, distribution, metabolism, excretion, and toxicity (ADMET) predictions were determined using the ADMET Predictor software 9.5 [63]. Final compounds are highly likely to cross the BBB (filter 98%), which is the same as for the previously described benzofuranyl-2-imidazole derivatives [28]. The percentage of unbound drugs in plasma is considered, with **LSL33** having the lowest value. P-glycoprotein (P-gp) is believed to play an important role in drug distribution and resistance to CNS drug treatment. The obtained results showed that none of the compounds studied were potential substrates for P-gp transporters or inhibitors of P-gp. Compounds are not human Ether-à-go-go-Related Gene (hERG) channel inhibitors, a major therapeutic challenge in drug discovery, except for **LSL35**. The results show that the final compounds have low cytochromes P450 (CYP) and TOX risk (Table 2).

The theoretical study does not give significant warnings, and therefore, in vitro experiments were undertaken, followed by in vivo studies.

### 3.3. Pharmacological Evaluation

#### 3.3.1. Blood-Brain Barrier Permeation Assay

“A good BBB-permeation is mandatory for developing new I_2_-IR ligands that show activity as neuroprotective agents." The in silico ADMET parameters are described in Section 3.2. indicate that final compounds are likely to cross the BBB. Therefore, the in vitro permeability (P_e_) of all the final compounds was determined by using the PAMPA-BBB assay (Table 3). "Considering the threshold established for high BBB permeation (P_e_ > 5.198 × 10^−6^ cm s^−1^), the studied compounds were well above this value”.

#### 3.3.2. Radioligand I_2_-IRs Binding Assays

The pharmacological profiles of six compounds (Figure 1C,D) were evaluated through competition binding studies against the selective I_2_-IR radioligand [^3^H]2-BFI and the selective α_2_-AR radioligand [^3^H]RX821002. The studies were performed on membranes from the *post-mortem* human frontal cortex, a brain area that shows an important density of I_2_-IRs and α_2_-AR. The inhibition constant (*K*_i_) for each compound was obtained and is expressed as the corresponding p*K*_i_. The selectivity for these two receptors was expressed by the I_2_/α_2_ index, calculated as the antilogarithm of the ratio between p*K*_i_ values for I_2_-IR and p*K*_i_ values for α_2_-AR. Competition experiments against [^3^H]2-BFI were biphasic for some of the compounds [64] (Table 4).

To determine the impact that the structural modifications that lead to the final compounds have on the affinity/selectivity data, we also consider the values of the standard compounds idazoxan, 2-BFI, CR4056 [65], and BU99008 [66], as well as the previously explored representative compound of the 2-imidazole-benzofurane family, LSL60101 [29] (Figure 1). We used as a reference compound idazoxan that showed an affinity for I_2_-IR (p*K*_i_ = 7.41 ± 0.63), with a p*Ki*_H_ I_2_ = 7.87 ± 0.74 (40% occupancy), a p*Ki*_L_ I_2_ = 5.76 ± 0.57 and an α_2_-AR (p*K*_i_ = 7.92 ± 0.07).

The standard I_2_-IR ligand 2-BFI depicted values of p*Ki*_H_ I_2_ = 9.08 ± 0.22 (*K*_i_ = 1 nM) and p*Ki*_L_ I_2_ = 7.15 ± 0.31 with 58% occupancy of the high affinity site, a p*Ki* I_2_ = 8.31 ± 0.13 when considering a monophasic curve, and a selectivity I_2_/α_2_ ratio of 5370. The clinical candidates CR4056 and BU99008 fitted best into a monophasic curve with values of p*Ki* I_2_ = 5.95 ± 0.11 and p*Ki* I_2_ = 7.05 ± 0.17, respectively, and negligible affinity upon α_2_-AR. The data found for LSL60101 fit best to a two-site model of binding with p*Ki*_H_ I_2_ = 8.17 ± 0.19 (*K*_i_ = 6.7 nM, 34% occupancy) and p*Ki*_L_ I_2_ = 6.02 ± 0.10 and an I_2_/α_2_ selectivity ratio of 3090. Fully aromatic compounds **LSL42** and **LSL39** showed the lowest affinity values (p*Ki* I_2_ = 5.24 ± 0.12 and p*Ki* I_2_ = 6.10 ± 0.25) of their congeners BU224 and idazoxan. Interestingly, the bioisoster of LSL60101 named **LSL33** depicted a high selectivity (p*Ki*_H_ I_2_ = 10.1 ± 0.57 (*K*_i_ = 79 pM) and p*Ki*_L_ I_2_ = 5.68 ± 0.25 with a 27% occupancy of the high site and low α_2_-AR affinity (p*Ki* I_2_ = 4.70 ± 0.12). Considering the modification that involves the *N*1-imidazole union to the heterocycle, we assessed the affinity/selectivity of **LSL35**, **LSL34**, and **LSL29**. Compound **LSL35** gave a similar value of affinity p*Ki* I_2_ = 5.49 ± 0.14 than its *N*2-imidazole partner **LSL42** and lower than BU224, indicating that the aromatization of the nitrogen five membered ring or the union by different positions to the heterocycle has no positive effect on the pharmacological activity. **LSL34** depicted improved affinity capabilities with respect to LSL60101, with a high affinity site that represents 27% occupancy and a p*Ki*_H_ I_2_ = 9.23 ± 0.36 (*K*_i_ = 59 nM) and a p*Ki*_L_ I_2_ = 5.68 ± 0.25 and low affinity upon α_2_-AR. The indol-derivative counterpart **LSL29** depicted interesting activity (p*Ki*_H_ I_2_ = 8.54 ± 0.71 and p*Ki*_L_ I_2_ = 4.09 ± 0.22) and a 22% high-site occupancy.

Compounds **LSL33** and **LSL34**, which showed the best pharmacological profile as ligands of the I_2_-IR in human tissues (Table 4), were tested in the I_2_-IR (antagonist radioligand) assay of Eurofins [67]. The competition binding assays were performed in rat cerebral cortex tissues employing [^3^H]idazoxan and 1 µM yohimbine to avoid α_2_-AR affinity of the non-selective radioligand. An inhibition higher than 50% is considered to represent significant effects of the test compounds, and **LSL33** showed an 84% inhibition and **LSL34** a 53% inhibition. These results confirm that **LSL33** and **LSL34** depicted activity as I_2_-IR ligands in both competition assays using different radioligands [^3^H]2-BFI and [^3^H]idazoxan and in two sources of tissues (human and rat).

### 3.4. 3D-QSAR Study

The created 3D-QSAR models were used to identify crucial structural features of the compounds that are important for I_2_-IR affinity and avoid α_2_-AR activity (Tables S2 and S3). The generated 3D-QSAR models were corroborated using the external and internal validation parameters listed in Tables S4 and S5. The validation results showed that both models can be used to predict the activity of the design molecules.

According to their chemical structures, all studied molecules can be classified into two clusters: Cluster I—containing imidazole and imidazoline derivatives—and Cluster II—consisting of bicyclic α-iminophosphonate derivatives previously described by us [23] (Figure S1).

The main variables that positively or negatively affect the activity of the compounds are represented by PLS coefficient plots (Figures S2 and S3).

**Figure 2:**
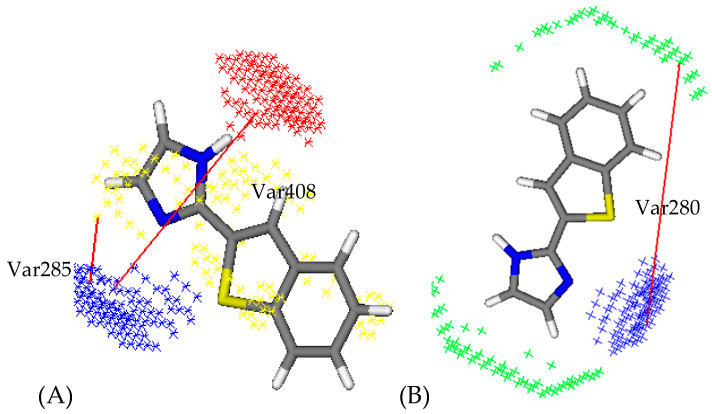
Favourable Var285: DRY-N1 and favourable Var408: O-N1 of compound **LSL33** ((**A**), 3D-QSAR (I_2_-IR) model); favourable Var280: DRY-N1 of **LSL33** ((**B**), 3D-QSAR (α_2_-AR) model); the hydrophobic regions (DRY) are labelled in yellow, H-bond donor regions in red, and H-bond acceptor regions in blue.

Among the synthesized compounds, the **LSL33** has favourable variables 285 (DRY–N1: 2Å–2.4Å) and 280 (DRY–N1: 4Å–4.4Å) describing the importance of the imidazole ring for the I_2_-IR and α_2_-AR activities (Figure 2). The favourable var285 is not described in the least active compound of the newly set **LSL29** or **LSL34**. Only these two molecules from this dataset have a heterocycle as a substituent on the nitrogen atom of the imidazole. The favourable variable 408 (O–N1: 6.4Å–6.8Å) is also present in the compound **LSL33** that is observed between nitrogen atoms of imidazole. Altogether, this indicates that the introduction of substituents on the nitrogen atom of the imidazole decreases the I_2_-IR activity.

The 3D-QSAR study, considering the compounds described in this paper and the previous I_2_-IR families, would allow us to propose future structural modifications to improve the pharmacological profile of newly designed I_2_-IR ligands (see Supplementary Materials for further details).

### 3.5. In Vitro Models of Neurodegenerative Diseases

#### 3.5.1. I_2_-IR Ligands Protects Neurons from Excitotoxic Damage

**Figure 3:**
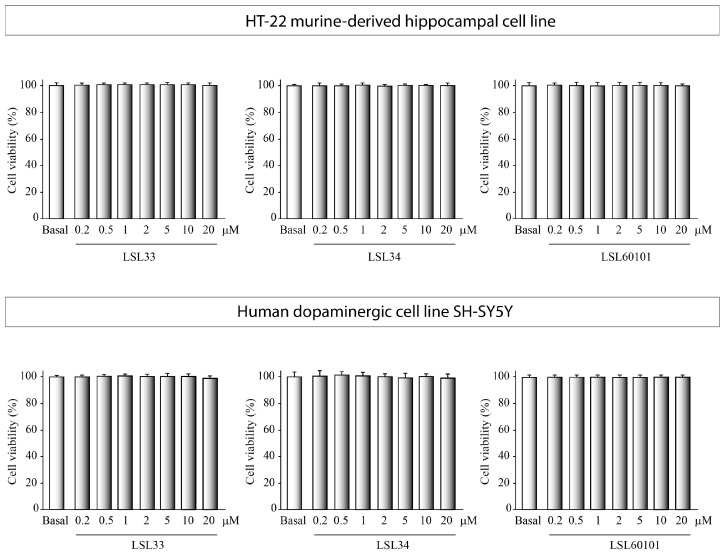
Analysis of the cytotoxicity of I_2_-IR ligands in vitro. HT-22 cells, derived from the murine hippocampus, and SH-SY5Y cells, representing human dopaminergic neurons, were exposed to escalating concentrations of the specified compounds for 24 h. The 3-(4,5-dimethylthiazol-2-yl)-5-(3-carboxymethoxyphenyl)-2-(4-sulfophenyl)-2*H*-tetrazolium (MTT) test was employed to assess the cytotoxic potential of the compounds. The reported values represent the mean ± SD obtained from triplicate determinations repeated a minimum of three times.

Both AD and PD share a common characteristic, namely the gradual reduction of specific neurotransmitters, such as acetylcholine in AD and dopamine in PD. This reduction stems from the degeneration of hippocampal neurons in AD and dopaminergic neurons in PD. Consequently, we have employed two widely recognized in vitro models of neuronal damage, which effectively replicate certain pathological events observed in these diseases. In order to evaluate the potential cytotoxic impact of the compounds themselves, cells were subjected to a range of concentrations. The HT-22 mouse hippocampal neuronal cell line was used as an in vitro model for investigating glutamate-induced neurotoxicity, which is closely associated with AD [68]. Similarly, the human neuronal dopaminergic cell line SH-SY5Y, owing to its shared characteristics with substantia nigra neurons, has been deemed a suitable in vitro system for simulating the properties of dopaminergic neurons [69]. Remarkably, all concentrations applied exhibited no detrimental effects on cellular viability (Figure 3).

**Figure 4:**
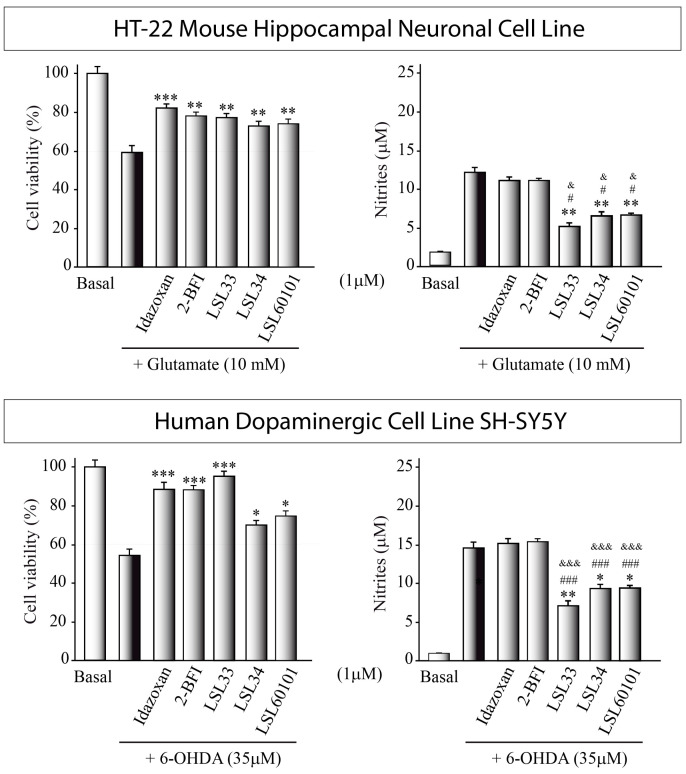
In vitro screening of I_2_-IR ligands: Assessment of neuroprotective and anti-inflammatory effects HT-22 cells exposed to 10mM glutamate for at least 16 h Similarly, SH-SY5Y was damaged with 35 μM 6-OHDA (35 μM). Some cultures were pretreated with the I_2_-IR ligands. Cell viability was evaluated using the MTT assay, and nitrite production in the cell supernatant was quantified using the Griess reaction. The reported values represent the mean ± SD obtained from triplicate determinations repeated at least three times. Statistical analysis was performed: * *p* ≤ 0.05, ** *p* ≤ 0.01, *** *p* ≤ 0.001 (versus glutamate- or 6-OHDA-treated cultures); ^#^ *p* ≤ 0.05, ^###^ *p* ≤ 0.001 (versus idazoxan-treated cultures); ^&^ *p* ≤ 0.1, ^&&&^ *p* ≤ 0.001 (versus 2-BFI-treated cultures).

Subsequently, we investigated whether I_2_-IR ligands possessed the ability to attenuate neuronal cell death induced by cytotoxic agents. Specifically, HT-22 hippocampal cells were exposed to 10 mM glutamate, while SH-SY5Y dopaminergic neurons were exposed to 35 μM 6-OHDA (Figure 4. For comparative purposes, the well-established I_2_-IR ligands idazoxan and 2-BFI were employed as standard treatments. Prior reports have documented the cytotoxicity analysis and effective dosage of idazoxan and 2-BFI [70]. Interestingly, our findings demonstrated that **LSL33**, **LSL34**, and LSL60101 exhibited potent neuroprotective effects in both cellular models of neurodegeneration, significantly mitigating toxin-induced neuronal cell death.

Together with the cytotoxic effect, glutamate in HT-22 hippocampal neurons and 6-OHDA in dopaminergic cells also induce a pronounced inflammatory response. Consequently, we proceeded to investigate the potential anti-inflammatory effects of the compounds by assessing nitrite generation in the culture supernatant (Figure 4). Treatment with **LSL33**, **LSL34**, and LSL60101 resulted in significant attenuation of the increased nitrite production induced by the toxin treatment, corroborating the observations from the cell viability assays and indicating a distinct anti-inflammatory effect of the compounds. Remarkably, I_2_-IR ligands exhibited superior anti-inflammatory properties compared to other I_2_-IR ligands, such as idazoxan and 2-BFI.

#### 3.5.2. I_2_-IR Ligands Decrease Neuroinflammatory Activity

In neurodegenerative diseases, neuroglia plays a critical role in the neuroinflammatory process. Glial cells, including microglia and astrocytes, become activated in response to various pathological stimuli in the brain, such as nitrosative and oxidative stress. This activation triggers a cascade of inflammatory responses, leading to the release of pro-inflammatory molecules and cytokines. Modulating glial cell activation and the associated inflammatory responses may offer potential avenues for neuroprotective interventions and disease modification in neurodegenerative diseases. Therefore, we proceeded to investigate whether the I_2_-IR ligands might be able to modify the inflammatory state in an in vitro model of neuroinflammation based on the use of two well-established glial cell lines, BV2 (microglia) and C6 (astroglia). Furthermore, primary cultures of microglial and astroglial cells derived from the mouse cortex were utilized to strengthen our research.

Glial cultures in the presence of a proinflammatory stimulus, such as LPS, become reactive glia, a state of activation and altered function that releases pro-inflammatory agents such as nitrites into the culture medium. Then, the measurement of nitrites serves as an indicator to evaluate the inflammatory state of the cultures.

**Figure 5:**
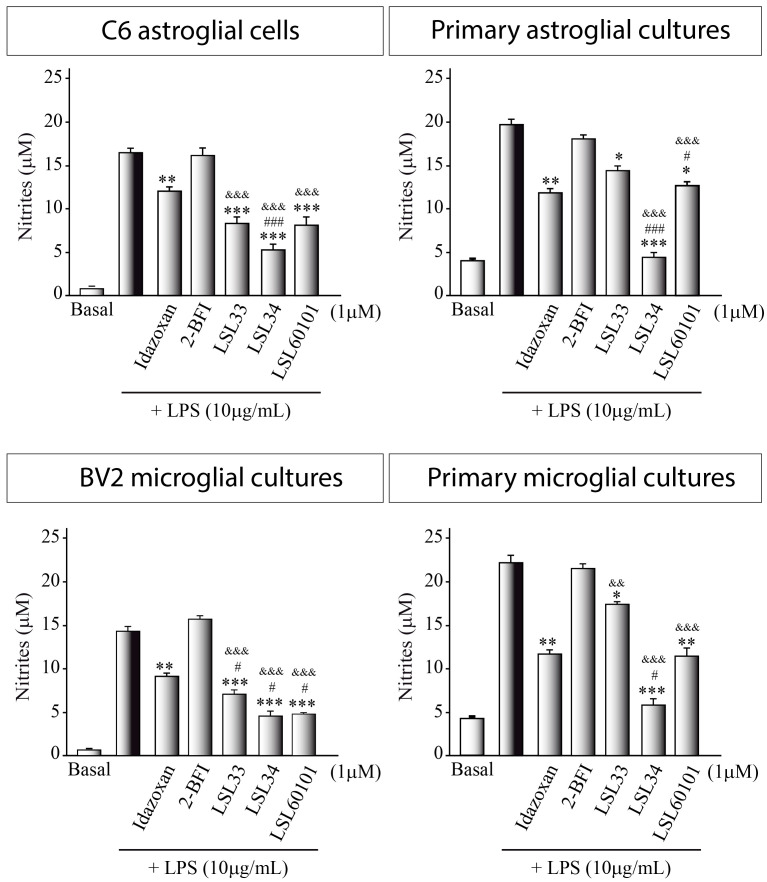
Role of I_2_-IR ligands in an in vitro model of neuroinflammation. Nitrite production in the supernatant of glial cells was assessed using the Griess reaction after a 24 h exposure to lipopolysaccharide (LPS, 10 μg/mL) in the presence of the indicated I_2_-IR ligands at a concentration of 1 μM. The values presented represent the mean ± SD from triplicate determinations, repeated at least three times. Statistical analysis revealed significant differences: * *p* ≤ 0.05 ** *p* ≤ 0.01, *** *p* ≤ 0.001 compared to LPS-treated cultures; ^#^ *p* ≤ 0.05, ^###^ *p* ≤ 0.001 compared to idazoxan-treated cultures; ^&&^ *p* ≤ 0.01, ^&&&^ *p* ≤ 0.001 compared to 2-BFI-treated cultures.

Figure 5 illustrates a notable reduction in nitrite levels, indicating a decrease in the pro-inflammatory state of LPS-stimulated cultures following treatment with I_2_-IR ligands. Intriguingly, this reduction surpasses the decrease observed with the standard ligands employed, underscoring the superior anti-inflammatory potential of these compounds. Taking into consideration that I_2_-IRs are widely distributed throughout the brain, particularly in glial cells [10], suggesting a plausible role in neuroinflammation, these results point to **LSL33**, **LSL34**, and LSL60101 as potential agents of therapeutic relevance in neuroinflammatory conditions associated with AD and PD.

### 3.6. Metabolic Stability of **LSL33** in Human Liver Microsomes

The efficacy and toxicity issues of a drug are directly related to its rate of elimination from the body. For this reason, the metabolic biotransformation of drug candidates is screened early in the discovery process. For example, the evaluation of the metabolic stability of drugs is performed using human liver microsomes (HLM).

The objective of this study was to evaluate the metabolic stability of **LSL33** and its depletion rate in the presence of HLM. This was achieved by incubating **LSL33** with microsomes and monitoring parent disappearance using LC/MSMS (Table S6).

For detailed experimental conditions see Supplementary Materials.

The percentage of the remaining parent compound and the time course of metabolic stability for **LSL33** and verapamil, the positive control, at each incubation time are displayed in Table 5, Figure S11, Table S7, and Figure S12, respectively.

The analysis of the percentage of **LSL33** remaining in minus NADPH cofactor incubations did not show the metabolism of the parent from non-dependent NADPH enzymes. The present study indicates that **LSL33** displays a half-life value equal to 1.58 min and a predicted intrinsic clearance of 422 mL/min/mg protein (Table S8). Attending to the classification bands typically used for categorizing compounds into low, medium, or high clearance (Table S9), **LSL33** showed hepatic clearance classified as high.

### 3.7. Pharmacokinetics of **LSL33**

The physicochemical and biochemical properties of **LSL33** have an enormous impact on its pharmacological behavior and determine the time course after its administration. The therapeutic characteristics of **LSL33** are determined by parameters such as the concentration in blood and tissues, distribution, metabolization, and elimination. Therefore, the pharmacokinetic (PK) profile of **LSL33** was investigated prior to the treatment of a murine model of AD (5xFAD). Due to the therapeutic indication addressed by **LSL33** in the CNS, we considered the quantity of compound that rises to brain tissues. We envisaged performing the cognitive studies after treating the animals with a dose of 2 mg/kg in drinking water for 4 weeks. Therefore, we considered a single oral administration of 2 mg/kg in CD1 mice for the PK study.

#### Pharmacokinetics of **LSL33** in Plasma

The resulting plasma profile and PK parameters of **LSL33** are shown in Table 6. For details on the method validation for quantification of **LSL33** in mouse plasma and in mouse brain (see Supplementary Materials).

The maximum concentration in plasma (C_max_) was 0.950 μg/mL ± 0.624 μg/mL and was reached at 15 min (T_max_). Plasma levels, although low, remain fairly constant for 8 h post-administration, and at 24 h, drug concentrations are almost undetectable. The half-life time (t_1/2_) was 312.8 min, and the AUC _0-_^1440^/AUC_0-_^∞^ ratio (0.97) shows that 24 h is a good interval to define the kinetic behavior of **LSL33** in plasma after oral administration at a dose of 2 mg/kg.

The maximum mean concentration found in plasma does not exceed 1% of the drug administered in a single oral dose. Notwithstanding the low levels, it should be noted that the administration of **LSL33** in drinking water at a dose of 2 mg/kg to the murine model (5xFAD) continued at effective levels for 4 weeks. These efficacy results could be explained by the elimination of half-life (312.81 ± 34.90 min), which will allow for reaching effective levels in the brain during treatment.

In the PK study, quantitation in mouse brain samples was considered, and after the single oral administration of 2 mg/kg at 15 min, a concentration of 15.2 ng/g was detected, and at 30 min, the concentration decreased to 6.7 ng/g. These data are in accordance with the BBB penetration properties envisaged in the PAMPA-BBB studies (Table 3).

### 3.8. Cognitive Studies

#### 3.8.1. **LSL33** Reduces Cognitive Impairment Presented in 5XFAD

Cognitive impairment is a clinical phenotype presented in AD human patients. To evaluate their working and spatial memories, mice were evaluated by the novel object recognition test (NORT) and the object location test (OLT), respectively. The NORT results revealed that 5XFAD treated with **LSL33** showed a significant improvement in cognitive impairment in both short- and long-term memories compared to the 5XFAD Control group, suggesting an amelioration in working memory.

**Figure 6:**
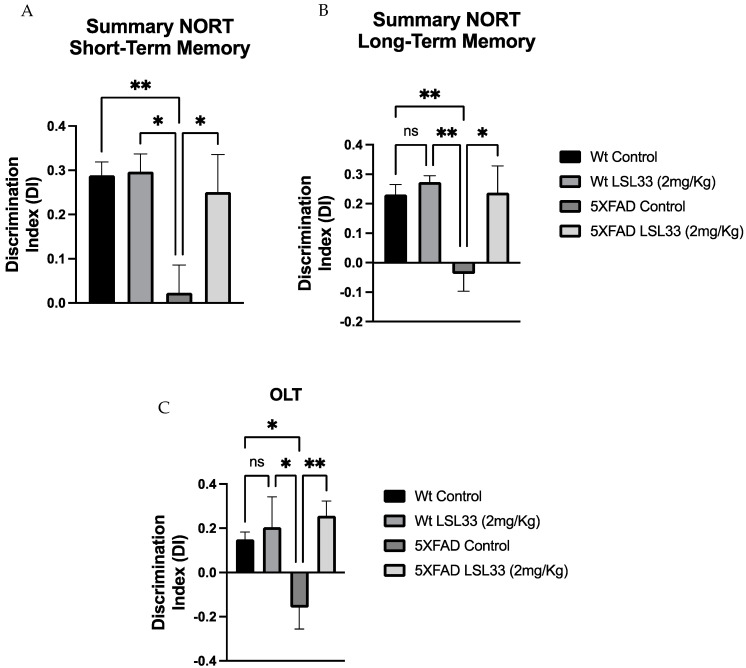
Results from cognition assays (**A**,**B**) Discrimination index of NORT of short- and long-term memories obtained in 7-month-old wild-type (WT) and 5XFAD mice, non-treated and treated for 4 weeks with **LSL33** at 2 mg/Kg, respectively. (**C**) OLT obtained in 7-month-old wild-type (WT) and 5XFAD mice, non-treated and treated for 4 weeks with **LSL33** at 2 mg/Kg, respectively. The data correspond to the mean ± SEM (*n* = 10–12 mice per group). Two-way ANOVA, Bonferroni’s post hoc test * *p* < 0.05, ** *p* < 0.01. ns: no significant.

Moreover, the OLT task results demonstrated that 5XFAD treated with **LSL33** exhibited an increase in the discrimination index (DI) compared to the 5XFAD Control, indicating an improvement in spatial memory after **LSL33** (2mg/Kg) treatment. Therefore, this data suggests that I_2_-IR ligands’ treatment strategy promotes beneficial effects on cognition in these AD transgenic mice (Figure 6).

#### 3.8.2. **LSL33** Ameliorates Synaptic Plasticity in 5XFAD

**Figure 7:**
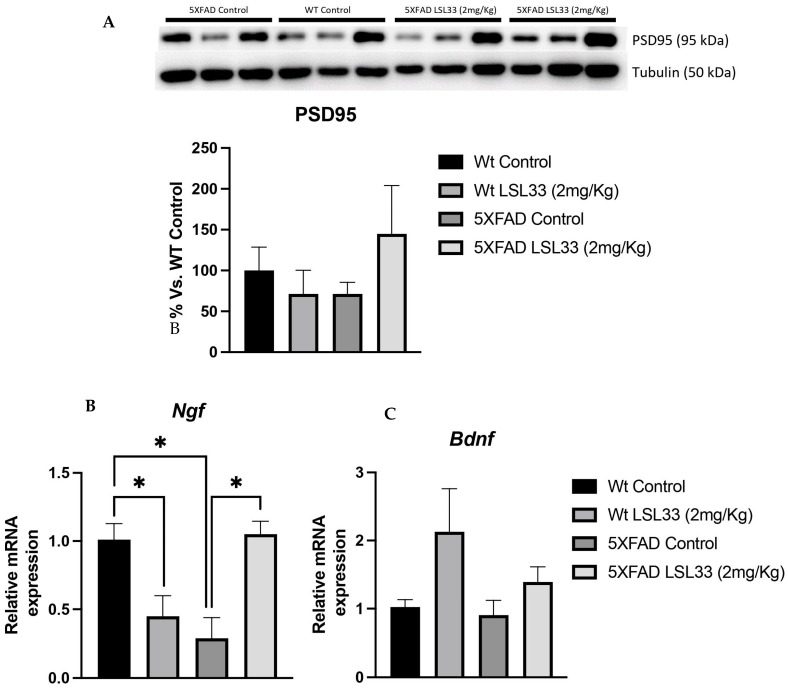
Results from an in vivo study of synaptic plasticity markers. (**A**) Representative protein levels and quantification by WB for PSD95. (**B**,**C**) Representative gene expression for *Ngf* and *Bdnf* Gene expression levels were determined by real-time PCR. The data correspond to the mean ± SEM (*n* = 3–5 mice per group). Two-way ANOVA and Bonferroni’s post hoc test; * *p* < 0.05.

Synaptic dysfunction is a symptomatic AD hallmark mainly associated with cognitive impairment [71]. Of note, the 5XFAD mouse model shows synaptic plasticity deficits as early as 2 months [39]. Thus, to further demonstrate the amelioration in synaptic plasticity after **LSL33** treatment in 5XFAD, we examined Postsynaptic density protein 95 (PSD95) protein levels and several neurotrophin gene expressions such as nerve growth factor (Ngf) and Brain derived-neurotrophic factor (Bdnf). Strikingly, an increase in PSD95 was observed in 5XFAD treated with I_2_-IR ligand (**LSL33**, 2 mg/Kg) in comparison with the 5XFAD Control mice (Figure 7A). Furthermore, as neurotrophins induce an increase in PSD95 [72] we evaluated Ngf and Bdnf. A significant increase in Ngf gene expression was found in 5XFAD treated with **LSL33** (2 mg/Kg) compared to the 5XFAD Control group (Figure 7B). Likewise, there was an increase in the Wt Control group in comparison with the Wt treated group, revealing a different pattern profile between strains (Figure 7C). Finally, we also evaluated Bdnf gene expression and only observed a clear tendency to increase in both treated groups with **LSL33** (2mg/Kg), suggesting the implication of I_2_-IRs in synaptic plasticity.

#### 3.8.3. **LSL33** Attenuates Neuroinflammation Presented in 5XFAD

**Figure 8:**
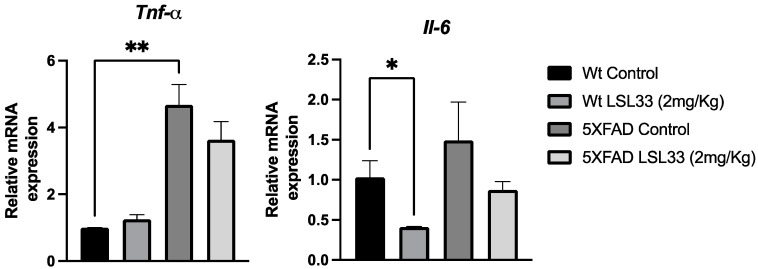
Results from an in vivo study Neuroinflammatory markers. Representative gene expression for *Tnf-a* and *Il-6* Gene expression levels were determined by real-time PCR. The data correspond to the mean ± SEM (*n* = 3–5 mice per group). Two-way ANOVA, Bonferroni’s post hoc test. * *p* < 0.05, ** *p* < 0.001.

One important AD hallmark is neuroinflammation, which is characterized by the release of several cytokines that contribute to disease progression and severity [73]. Then, we determined by qPCR two of the main pro-inflammatory mediators, such as tumour necrosis factor-alpha (Tnf-a) and Interleukin 6 (Il-6). Firstly, we found an increase in both gene expression levels in the 5XFAD Control group compared to the Wt Control group (Figure 8). Interestingly, we found a clear tendency to reduce both cytokines in 5XFAD treated with **LSL33** (2 m/Kg) in comparison with 5XFAD Control, suggesting that I_2_-IRs modulate neuroinflammation (Figure 8).

## 4. Conclusions

There is a consensus amongst the scientific community that believes that to overcome AD, new therapeutic targets need to be explored and modified to find an amelioration or eventually a cure. In this respect, we are exploring the pharmacological anti-AD effects of modulating I_2_-IRs. In this work, we deliver molecular entities that result from the observation/combination of key structural features of standard and well-established I_2_-IR ligands. The final molecules displayed good brain permeation, were submitted to affinity/selectivity I_2_-IR competition studies, permitted the proposal of a pharmacophore after 3D-QSAR, and the theoretical ADME and physicochemical parameters were calculated to rule out warnings to continue with the medicinal chemistry program. Representative compounds were chosen to study their neuroprotective role in the human dopaminergic cell line SH-SY5Y. Furthermore, in primary cultures of astrocytes and microglia treated with LPS, selected compounds were shown to be anti-inflammatory agents even better than those of other known ligands. A preliminary pharmacokinetic study was performed with **LSL33** to continue with its further preclinical development. Taking into account the improvement in cognitive impairment in a 5xFAD model treated with **LSL33**, modulation of I_2_-IRs can be proposed as a new therapeutic strategy for AD treatment.

## Figures and Tables

**Table 1:** Results of physicochemical parameters of **LSL42**, **LSL39**, **LSL33**, **LSL35**, **LSL34**, and **LSL29** obtained with the SwissADME web tool.

Compound	Log S (ESOL) −10 to 0	Solubility Class	Log P_o/w_ (XLOGP3 < 5)	Log P_o/w_ (MLOGP < 4.15)	Log P_o/w_ (WLOGP < 5.6)	Rule Of5	TPSA 20Å^2^ < TPSA < 130
**LSL42**	−3.00	Soluble	2.03	1.39	2.62	0	41.57
**LSL39**	−2.41	Soluble	1.34	0.46	1.60	0	47.14
**LSL33**	−3.51	Soluble	2.78	2.03	3.29	0	56.92
**LSL35**	−3.15	Soluble	2.27	1.39	2.42	0	30.71
**LSL34**	−3.17	Soluble	2.41	1.15	2.62	0	30.96
**LSL29**	−2.99	Soluble	2.13	1.55	2.35	0	33.61

Legend: Log S (ESOL)—water solubility, topological method implemented from Delaney Model [60]; Solubility Class—insoluble < −10 < poorly < −6 < moderately < −4 < soluble < −2 < very < 0 < highly; XLOGP3—atomistic and knowledge-based method calculated by the XLOGP program; MLOGP—topological method implemented by Moriguchi I. [61], WLOGP—atomistic method implemented by Wildman S. A. and Crippen G. M. [62].; RuleOf5—Lipinski’s “Rule of Five”; TPSA—topological polar surface area.

**Table 2:** Results of ADMET properties obtained with ADMET Predictor software 9.5.

Compound	BBB Filter	Hum_Fup%	Pgp_Substr	Pgp_Inh	CYP_Risk	TOX_Risk	hERG_Filter
**LSL42**	High (98%)	10.35	No (93%)	No (96%)	0.15	1.00	No (54%)
**LSL39**	High (98%)	25.17	No (93%)	No (96%)	0.28	0	No (96%)
**LSL33**	High (98%)	6.06	No (93%)	No (96%)	0.47	1.00	No (77%)
**LSL35**	High (98%)	10.85	No (93%)	No (96%)	0.72	1.00	Yes (54%)
**LSL34**	High (98%)	10.66	No (93%)	No (81%)	1.10	1.00	No (96%)
**LSL29**	High (98%)	13.81	No (93%)	No (96%)	0.38	1.04	No (47%)

Legend: BBB filter—qualitative likelihood (High/Low) of crossing the BBB; hum_fup%—percentage of unbound drug in plasma; Pgp_Substr—likelihood of P-glycoprotein efflux; Pgp_Inh—likelihood of a molecule being an inhibitor of P-glycoprotein; CYP_Risk—cytochrome P450 liability score; Tox_Risk—toxicity liability score; hERG_Filter—qualitative estimation of the likelihood of hERG potassium channel inhibition in humans.

**Table 3:** Permeability results (P_e_ 10^−6^ cm s^−1^) from the PAMPA-BBB assay for final compounds and their prediction of BBB permeation.

Compound	^a^ Permeability P_e_ (10^−6^ cm·s^−1^)	^b^ Prediction
**LSL60101**	13.6 ± 0.4	CNS+
**LSL42**	5.8 ± 0.2	CNS+
**LSL33**	8.1 ± 0.4	CNS+
**LSL35**	16.2 ± 0.9	CNS+
**LSL34**	14.1 ± 0.5	CNS+
**LSL29**	10.8 ± 0.4	CNS+

^a^. PBS/EtOH (70:30) was used as a solvent. Values are expressed as the mean ± SD of at least three independent experiments with *n* = 3 in each experiment. ^b^. The in vitro permeability (Pe) of fourteen commercial drugs through a lipid extract of porcine brain membrane together with the test compounds was determined (for the commercial drug values, see Table S1).

**Table 4:** I_2_-IR and α_2_-AR binding affinities (p*K*_i_) of compounds idazoxan, 2-BFI, CR4056, BU99008, LSL60101 and compounds **LSL42**, **LSL39**, **LSL33**, **LSL35**, **LSL34** and **LSL29**.

Compound	^a^ [^3^H]-2-BFI I_2_ p*K*i (One Site)	^b^ [^3^H]-2-BFI I_2_ p*K*i (Two Sites)		High-Affinity Site (% Occupancy)	[^3^H]-RX821002 α_2_ p*K*i	Selectivity I_2_/α_2_
**Idazoxan**	7.41 ± 0.63	7.87 ± 0.74	5.76 ± 0.57	40 ± 7	7.92 ± 0.07	-
**2-BFI**	8.31 ± 0.13	9.08 ± 0.22	7.15 ± 0.31	58 ± 9	4.58 ± 0.22	5370
**CR4056**	5.95 ± 0.11				2.65 ± 1.24	1995
**BU99008**	7.05 ± 0.17				4.37 ± 0.17	479
**LSL60101**	6.67 ± 0.09	8.17 ± 0.19	6.02 ± 0.10	34 ± 4	3.18 ± 0.17	3090
**LSL42**	5.24 ± 0.12	-	-	-	5.32 ± 0.24	
**LSL39**	6.10 ± 0.25	-	-	-	3.97 ± 0.25	
**LSL33**	5.95 ± 0.24	10.1 ± 0.57	5.68 ± 0.25	27 ± 6	4.70 ± 0.12	
**LSL35**	5.49 ± 0.14	-	-	-	5.24 ± 0.26	
**LSL34**	5.58 ± 0.31	9.23 ± 0.36	4.64 ± 0.32	37 ± 6	5.40 ± 0.21	
**LSL29**	4.57 ± 0.23	8.54 ± 0.71	4.09 ± 0.22	22 ± 5	4.99 ± 0.18	

^a^ Selectivity I_2_-IR/α_2_-AR expressed as the antilog (p*K*_i_ I_2_-IR − p*K*_i_ α_2_-AR). ^b^ The best fit of the data for most of the compounds was to a two-site binding model with high p*K*_i_ (p*K*_iH_) and low p*K*_i_ (p*K*_iL_) affinities for both binding sites, respectively.

**Table 5:** Percentage of **LSL33** remaining at each incubation time in HLM.

Time (min)	% Compound Remaining
	Plus NADPH
0	100.00 ± 9.0
5	4.6 ± 0.4
15	0.1 ± 0.1
30	0.2 ± 0.1
45	1.3 ± 0.2
60	0.2 ± 0.3
	Minus NADPH
0	100.0 ± 10.2
60	96.6 ± 9.0

**Table 6:** Basic PK parameters were calculated. C_max_: maximum observed concentration; T_max_: time of maximum observed concentration; t_1/2β_: terminal elimination half-life; AUC_0-_^1440^: area under the curve from zero to the last sampling time; AUC_0-_^∞^: area under the curve from zero extrapolated to infinity; CI: plasma clearance; Vz: volume of distribution. For experimental details on the method validation for quantification of **LSL33** in mouse plasma/brain see Supplementary Materials.

Pharmacokinetic Parameters	
t_1/2β_ (min)	312.81 ± 34.90
T_max_ (min)	15 ± 0
C_max_ (μg/mL)	0.95 ± 0.62
AUC_0-_^1440^ (μg/mL·min)	176.98 ± 37.78
AUC_0-_^∞^ (μg/mL·min)	182.99 ± 38.07

## Data Availability

The data presented in this study are available in this article.

## Supplementary Materials

The following Supplementary Materials can be downloaded at: https://www.mdpi.com/article/10.3390/pharmaceutics15102381/s1, ^1^H and ^13^C spectra of representative compounds, in vitro PAMPA-BBB, 3D-QSAR study, metabolic stability of **LSL33** in HLM, Methods validation for quantification of **LSL33** in mouse plasma and brain, antibodies for Western blood, and Syber green primers used in qPCR studies. References [74,75,76,77] are cited in the supplementary materials.
