# Supplementary material for: Preclinical Evaluation of an Imidazole-Linked Heterocycle for Alzheimer’s Disease
Source: Pharmaceutics. 2023 Sep 25;15(10):2381. doi: 10.3390/pharmaceutics15102381 (PMC10610545; doi:10.3390/pharmaceutics15102381)
Supplement: Supplementary file 1 [file pharmaceutics-15-02381-s001.zip › pharmaceutics-2550485-supplementary.pdf]

Preclinical evaluation of an imidazole linked heterocycle for Alzheimer's Disease

Andrea Bagán[1], Sergio Rodriguez-Arévalo[1], Teresa Taboada-Jara[2], Christian Griñán-Ferré[2,3], Mercè Pallàs[2,3], Iria Brocos-Mosquera[4], Luis F. Callado[4,5], José A. Morales-García[6], Belén Pérez[7], Caridad Diaz[8], Rosario Fernández[8], Olga Genilloud[8], Milan Beljkas[9], Slavica Oljacic[9], Katarina Nikolic[9] and Carmen Escolano[1],*

[1] Laboratory of Medicinal Chemistry (Associated Unit to CSIC), Department of Pharmacology, Toxicology and Medicinal Chemistry, Faculty of Pharmacy and Food Sciences, and Institute of Biomedicine (IBUB), University of Barcelona, Av. Joan XXIII, 27-31, E-08028, Barcelona.
[2] Pharmacology Section, Toxicology and Medicinal Chemistry, Faculty of Pharmacy and Food Sciences, and Institut de Neurociències, University of Barcelona, Av. Joan XXIII, 27-31. E-08028, Barcelona, Spain.
[3] Centro de Investigación Biomédica en Red Enfermedades Neurodegenerativas (CiberNed), National Institute of Health Carlos III, 28029 Madrid, Spain.
[4] Department of Pharmacology, University of the Basque Country, UPV/EHU, E-48940 Leioa, Bizkaia, and Centro de Investigación Biomédica en Red de Salud Mental, CIBERSAM, Spain.
[5] Biocruces Bizkaia Health Research Institute, Barakaldo, Spain.
[6] Department of Cell Biology, School of Medicine, Complutense University (UCM), 28040 Madrid, Spain.
[7] Department of Pharmacology, Therapeutic and Toxicology. Autonomous University of Barcelona, E-08193 Cerdanyola. Spain.
[8] Fundación MEDINA Centro de Excelencia en Investigación de Medicamentos Innovadores en Andalucía, Avda. del Conocimiento 34, 18016 Armilla, Spain.
[9] Department of Pharmaceutical Chemistry, Faculty of Pharmacy, University of Belgrade, Vojvode Stepe 450, 11221 Belgrade, Serbia.

CONTENTS

General information for the synthetic reactions **S2**

^{1}H-NMR and ^{13}C-NMR spectra of representative compounds **S3-S16**

Preparation of cellular membranes **S17**

Competition binding assays **S17**

***In vitro* Blood-Brain Barrier Permeation Assay** **S18**

3D-QSAR study **S19-S30**

Animals for PK studies **S31**

Metabolic stability of LSL33 in human liver microsomes **S32**

Method validation for quantification of LSL33 in Mouse Plasma **S34**

Method validation for quantification of LSL33 in Mouse Brain **S38**

Antibodies used in Western blot studies **S42**

Syber green primers used in qPCR studies **S42**

General information.

Reagents, solvents and starting products were acquired from commercial sources. The term "concentration" refers to the vacuum evaporation using a Büchi rotavapor. When indicated, the reaction products were purified by "flash" chromatography on silica gel (35-70 μm) with the indicated solvent system. IR spectra were performed in a Spectrum Two FT-IR Spectrometer, and only noteworthy IR absorptions (cm^{-1}) are listed. NMR spectra were recorded in DMSO-*d*6 or CD_3OD at 400 MHz (^1H) and 100.6 MHz (^{13}C), and chemical shifts are reported in δ values downfield from TMS or relative to residual DMSO-*d*6 (2.50 ppm, 39.5 ppm) or CD_3OD (3.31 ppm) as an internal standard. Data are reported in the following manner: chemical shift, multiplicity, coupling constant (*J*) in hertz (Hz) and integrated intensity. Multiplicities are reported using the following abbreviations: s, singlet; d, doublet; dd, doublet of doublets; t, triplet; m, multiplet; br s, broad signal. The accurate mass analyses were carried out using a LC/MSD-TOF spectrophotometer. HPLC-MS (Agilent 1260 Infinity II) analysis was conducted on a Poroshell 120 EC-C15 (4.6 mm × 50 mm, 2.7 μm) at 40 °C with mobile phase A (H_2O + 0.05 % formic acid) and B (ACN + 0.05 % formic acid) using a gradient elution and flow rate 0.6 mL/min. The DAD detector was set at 254 nm, the injection volume was 5 μL, and oven temperature was 40 °C. All tested compounds possess a purity of at least 95 %.

Preparation of cellular membranes.

Human brain samples were obtained at autopsy in the Basque Institute of Legal Medicine, Bilbao, Spain. Samples from the prefrontal cortex (Brodmann's area 9) were dissected at the time of autopsy and immediately stored at -70 °C until assay. The study was developed in compliance with policies of research and ethical review boards for postmortem brain studies.

To obtain cellular membranes (P2 fraction) the different samples were homogenized using an ultraturrax in 10 volumes of homogenization buffer (0.25 M sucrose, 5 mM Tris–HCl, pH 7.4). The crude homogenate was centrifuged for 5 min at 1000 g (4 °C) and the supernatant was centrifuged again for 10 min at 40,000 g (4 °C). The resultant pellet was washed twice in 5 volumes of homogenization buffer and recentrifuged in similar conditions. Protein content was measured according to the method of Bradford using BSA as standard.

Competition Binding Assays.

The pharmacological activity of the compounds was evaluated through competition binding studies against the I_2-IR selective radioligand [^{3}H]2-BFI or the α_2-adrenergic receptor selective radioligand [^{3}H]RX821002. Specific binding was measured in 0.25 mL aliquots (50 mM Tris-HCl, pH 7.5) containing 100 μg of membranes, which were incubated in 96-well plates either with [^{3}H]2-BFI (2 nM) for 45 min at 25 °C or [^{3}H]RX821002 (1 nM) for 30 min at 25 °C, in the absence or presence of the competing compounds (10^{-12} to 10^{-3} M, 10 concentrations).

Incubations were terminated by separating free ligand from bound ligand by rapid filtration under vacuum (1450 Filter Mate Harvester, PerkinElmer) through GF/C glass fiber filters. The filters were then rinsed three times with 300 μL of binding buffer, air-dried (120 min), and counted for radioactivity by liquid scintillation spectrometry using a MicroBeta TriLux counter (PerkinElmer). Specific binding was determined and plotted as a function of the compound concentration. Nonspecific binding was determined in the presence of idazoxan (10^{-5} M), a compound with well stablished affinity for I_2-IR and α_2-adrenergic receptors, in [^{3}H]2-BFI and [^{3}H]RX821002 assays. To obtain the inhibition constant (K_i) analyses of competition experiments were performed by nonlinear regression using the GraphPad Prism program. K_i values were normalized to pK_i values. I_2-IR/α_2 selectivity index was calculated as the antilogarithm of the difference between pK_i values for I_2-IR and pK_i values for α_2-ARs.

In vitro **Blood-Brain Barrier Permeation Assay**

To evaluate the brain penetration of the different compounds, a parallel artificial membrane permeation assay for blood-brain barrier was used, following the method described by Di et al.[1] The *in vitro* permeability (P_e) of fourteen commercial drugs (see **Table S1**) through lipid extract of porcine brain membrane together with the test compounds were determined. Commercial drugs and assayed compounds were tested using a mixture of PBS:ETOH (70:30). Assay validation was made by comparing the experimental permeability with the reported values of the commercial drugs by bibliography and lineal correlation between experimental and reported permeability of the fourteen commercial drugs using the parallel artificial membrane permeation assay was evaluated (y = 1.572x-1.090; R^2=0.938). From this equation and taking into account the limits established by Di et al. for BBB permeation [1], we established the ranges of permeability as compounds of high BBB permeation (CNS+): *P*e (10^{-6} cms^{-1}) > 5.198; compounds of low BBB permeation (CNS-): *P*e (10^{-6} cm s^{-1}) < 2.054 and compounds of uncertain BBB permeation (CNS+/-): 5.198 > *P*e (10^{-6} cm s^{-1}) > 2.054.

Table S1. Permeability (*Pe* 10^{-6} cm s^{-1}) in the PAMPA-BBB assay of the 14 commercial drugs predictive penetration in the CNS used as references.

**Compound**	**Bibliography value[(a)]**	**Experimental value (n=3) ± S.D.**	***CNS Prediction***
Verapamil	16.0	25.4±0.6	*CNS*+
Testosterone	17.0	27.1±0.5	CNS+
Costicosterone	5.1	6.7±0.1	CNS+
Clonidine	5.3	6.5±0.05	CNS+
Ofloxacin	0.8	0.1±0.08	CNS-
Lomefloxacin	0.0	0.85±0.03	CNS-
Progesterone	9.3	16.8±0.3	CNS+
Promazine	8.8	13.8±0.3	CNS+
Imipramine	13.0	12.5±0.2	CNS+
Hidrocortisone	1.9	1.4±0.05	CNS-
Piroxicam	2.5	1.9±0.07	CNS-
Desipramine	12.0	17.8±0.1	CNS+
Cimetidine	0.0	0.7±0.03	CNS-
Norfloxacine	0.1	8.8±0.5	CNS+

[1] Di, L.; Kerns, E. H.; Fan, K.; McConnell, O. J.; and Carter, G. T. High throughput artificial membrane permeability assay for blood-brain barrier. *Eur. J. Med. Chem.* **2003**, *38*, 223-232.

3D-QSAR study

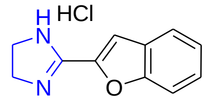
2-BFI

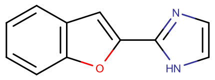
LSL60101

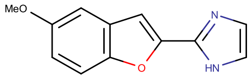
LSL60131

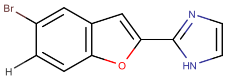
LSL60121

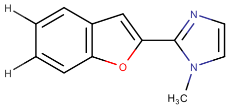
LSL60111

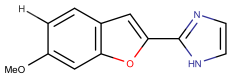
LSL60125

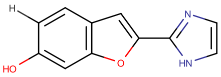
LSL60128

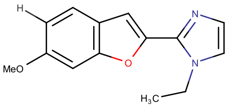
LSL60119

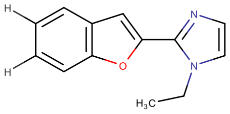
LSL60112

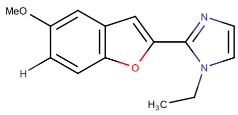
LSL13

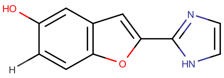
LSL60133

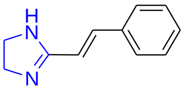
Tracizoline

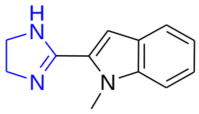
BU99008

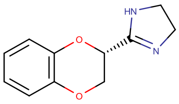
Idazoxan

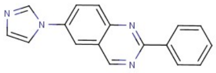
CR4056

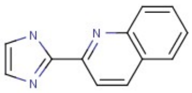
LSL42

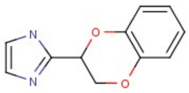
LSL39

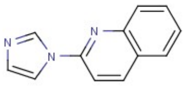
LSL35

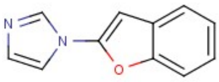
LSL34

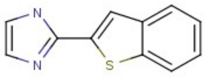
LSL33

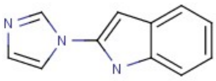
LSL29

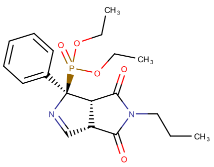
B51

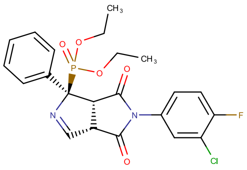
B06

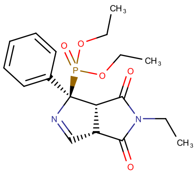
B54

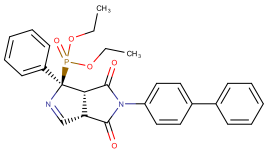
B32

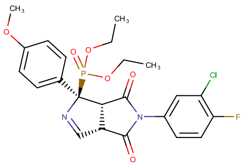
B64

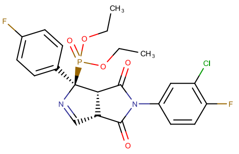
B62

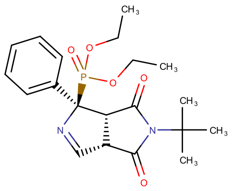
B55

Figure S1. Chemical structures of studied compounds

Table S2. Distribution of compounds in the training and test set along with the experimentally obtained and the pK_i (I_2) values predicted by the created 3D-QSAR (I_2-IR) model.

	Training set		Test set		
	**pK_i (exp)**	**pK_i (pred)**		**pK_i (exp)**	**pK_i (pred)**
**B51**	4.020	4.877	**LSL13**	4.960	5.195
**LSL29**	4.570	5.116	**LSL60119**	5.260	5.359
**LSL60133**	4.870	4.693	**LSL35**	5.490	4.821
**LSL60112**	5.050	5.461	**LSL60125**	5.880	5.499
**LSL42**	5.240	5.385	**LSL60121**	6.280	6.357
**LSL60128**	5.480	5.606	**LSL60101**	6.670	6.345
**LSL34**	5.580	4.772	**B55**	7.350	7.505
**LSL33**	5.950	5.832	**Tracizoline**	7.580	7.107
**CR4056**	5.950	6.168	**B32**	7.900	6.701
**LSL60111**	5.980	5.543			
**LSL39**	6.100	5.988			
**B33**	6.350	5.940			
**LSL60131**	6.410	6.056			
**B22**	6.650	6.755			
**B34**	6.960	7.246			
**BU99008**	7.050	7.564			
**idazoxane**	7.410	7.253			
**B62**	7.550	7.413			
**B64**	7.870	.7.359			
**2BFI**	8.310	8.272			
**B54**	8.370	7.625			
**B06**	8.560	8.549			

Table S3. Distribution of compounds in the training and test set along with the experimentally obtained and the p*K*i (α_2) values predicted by the created 3D-QSAR (α_2-AR) model.

**Training set**			**Test set**		
	**pK_i (exp)**	**pK_i (pred)**		**pK_i (exp)**	**pK_i (pred)**
**CR4056**	2.650	2.867	**LSL60119**	3.170	4.054
**LSL60125**	3.010	3.193	**LSL60128**	3.760	3.386
**LSL60101**	3.180	3.938	**LSL60133**	3.840	3.533
**B62**	3.380	3.159	**LSL13**	4.210	4.226
**LSL60111**	3.750	3.597	**Tracizoline**	4.330	4.477
**LSL60112**	3.770	3.352	**LSL29**	4.990	6.109
**B33**	3.770	3.650	**LSL35**	5.240	4.459
**LSL60131**	3.940	3.702	**B34**	5.430	5.328
**LSL39**	3.970	3.791	**B55**	6.770	6.673
**BU99008**	4.370	4.578			
**2BFI**	4.580	5.089			
**B22**	4.590	4.735			
**LSL33**	4.700	4.543			
**LSL60121**	4.920	4.850			
**B32**	5.120	5.223			
**LSL42**	5.320	4.812			
**LSL34**	5.400	5.125			
**B54**	5.850	5.908			
**B06**	6.270	6.633			
**idazoxane**	7.920	7.528			

Internal and external validation of 3D-QSAR models

The internal predictive ability and robustness of the developed 3D-QSAR models are tested by the following parameters: R^2 (coefficient of determination) (Eq.1), Q^2LOO (Leave-One-Out Cross-Validated squared correlation coefficient) (Eq.2), and RMSEE (Root Mean Square Error of Estimation) (Eq.3). For a predictive QSAR models, values of Q^2 should be higher than 0.5.[2,3]

$$R^2 = \frac{\Sigma\left(\hat{Y}_{training} - \bar{Y}_{training}\right)^2}{\Sigma(Y_{training} - \bar{Y}_{training})^2} \quad (1)$$

$$Q^2 = 1 - \frac{PRESS}{\Sigma(Y_{training} - \bar{Y}_{training})^2} \quad (2)$$

$$RMSEE = \sqrt{\frac{PRESS}{n}} \quad (3)$$

The external validation of 3D-QSAR models is evaluated by test set. The following parameters were used for external validation: R^2_{pred}, RMSEP (Root Mean Square Error of Prediction), and r^2_m metrics[4] calculated using the following equations (Eq.4-7):

$$R^2_{pred} = 1 - \frac{\Sigma\left(Y_{test} - \hat{Y}_{test}\right)^2}{\Sigma\left(Y_{test} - \bar{Y}_{training}\right)^2} \quad (4)$$

$$RMSEP = \sqrt{\frac{PRESS}{n}} \quad (5)$$

$$r^2_m = r^2(1 - \sqrt{r^2 - r^2_0}) \quad (6)$$

$$r^{/2}_m = r^2(1 - \sqrt{r^2 - r^{/2}_0}) \quad (7)$$

[2] Tropsha, A.; Best Practices for QSAR Model Development, Validation, and Exploitation. *Mol. Inform.* **2010**, 29, 476.
[3] Golbraikh, A.; Tropsha, A. Beware of q2. *J. Mol. Graph. Model.* **2002**, 20, 269.
[4] Roy, K.; Mitra, I.; Kar, S.; Ojha, P. K.; Das, R. N.; Kabir, H. Comparative studies on some metrics for external validation of QSPR models. J. Chem. Inf. Model. 2012, 52, 396–408.

Table S4. Results of developed 3D-QSAR (I_2-IR) model

**Internal validation parameters**				
	**R^2**	**Q^2_{LOO}**	**RMSEE**	
	**0.892**	**0.61**	**0.410**	
Criteria	>0.7	>0.5		

**External validation parameters**				
	**R^2_{pred}**	**RMSEP**	**r_m^2**	**r'^2_m**
	**0.730**	**0.522**	**0.800**	**0.643**
Criteria	>0.6	≤2RMSEE	>0.5	>0.5
	$\overline{r_m^2}$	**Δr^2_m**	**$(r^2-r'^2_0)/r^2$**	**k'**
	**0.721**	**0.157**	**0.051**	**0.952**
Criteria	>0.5	<0.2	<0.1	0.85 ≤ k' ≤ 1.15

Table S5. Results of developed 3D-QSAR (α_2-AR) model

**Internal validation parameters**				
	**R^2**	**Q^2_{LOO}**	**RMSEE**	
	**0.919**	**0.62**	**0.315**	
Criteria	>0.7	>0.5		

**External validation parameters**				
	**R^2_{pred}**	**RMSEP**	**r_m^2**	**r'^2_m**
	**0.693**	**0.570**	**0.609**	**0.637**
Criteria	>0.6	≤2RMSEE	>0.5	>0.5
	$\overline{r_m^2}$	**Δr^2_m**	**$(r^2-r'^2_0)/r^2$**	**k'**
	**0.623**	**0.027**	**0.021**	**1.006**
Criteria	>0.5	<0.2	<0.1	0.85 ≤ k' ≤ 1.15

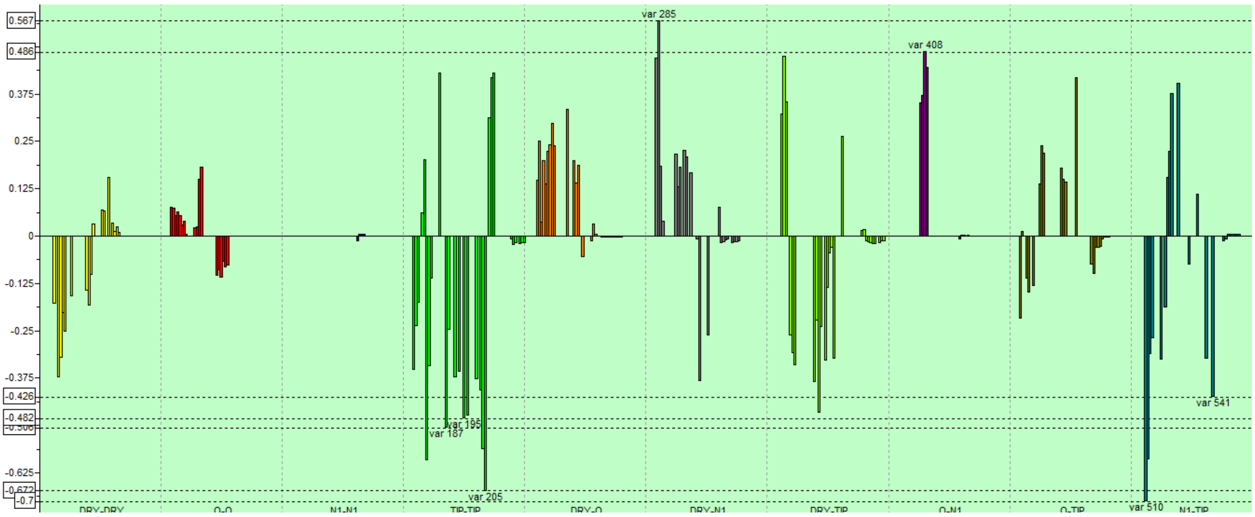

Figure S2. PLS coefficient plot for the 3D-QSAR (I_2-IR) model. The most significant variables are labelled.

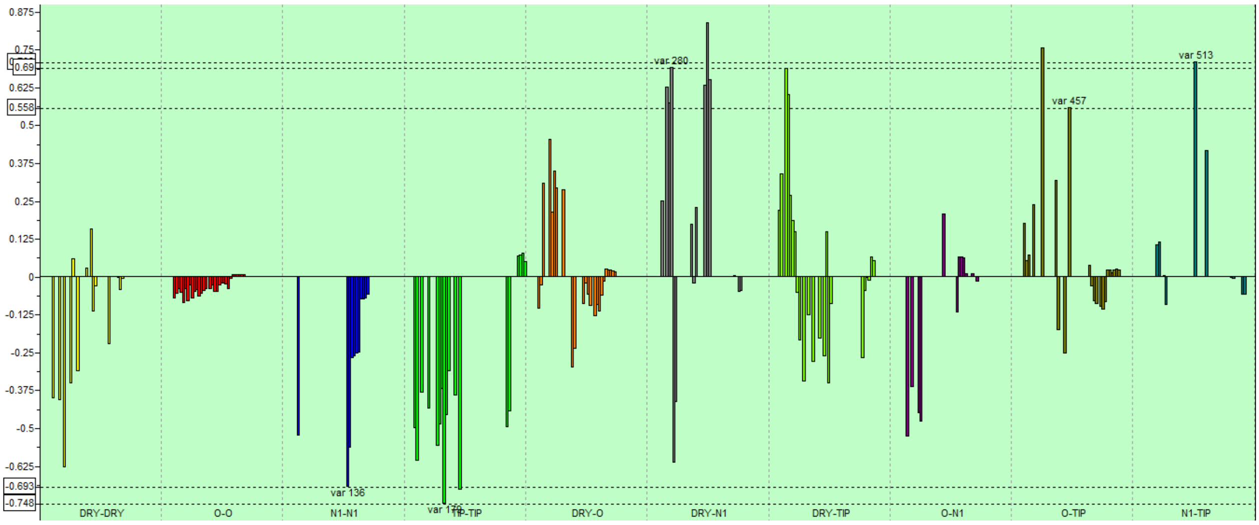

Figure S3. PLS coefficient plot for the 3D-QSAR (α_2-AR) model. The most significant variables are labelled

In the both 3D-QSAR models the favourable var 285 (DRY -N1: 2-2.4A) and the favourable var 280 (DRY-N1: 4A-4.4A) described between the hydrogen accepting group (nitrogen atom of imidazole and imidazoline) and the hydrophobic region around imidazole ring hot spot are presented in all molecules of the cluster I. It indicates that this part of molecule is important for the I_2-IR and α_2-AR activity but does not have the impact on the selectivity of compounds (Figure S4).

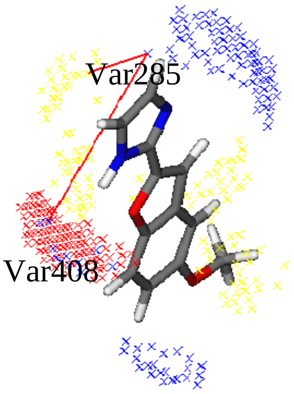

Figure S4. Favourable Var285: DRY-N1 and favourable Var408: O-N1 of compound LSL60131 (3D-QSAR (I_2-IR) model); the hydrophobic regions (DRY) are labeled in yellow, H-bond donor regions in red and H-bond acceptor regions in blue.

The favourable var 408 (O-N1: 6.4 - 6.8 Å) describing the distance between the hydrogen bond acceptor and hydrogen bond donor probes around the nitrogen atoms of imidazole shows the importance of the unsubstituted imidazole ring for I_2-IR activity (Figure S4). Moreover, the unfavourable var 187 (TIP-TIP: 7.6 Å - 8 Å) and the unfavourable var179 (TIP-TIP: 6.8 Å – 7.2 Å) underline the negative influence of the distance between the steric regions around imidazole and its substituents on the nitrogen atom on the I_2-IR activity and α_2-AR activity (LSL6011, LSL60112) (Figure S5). These results are consistent with the experimental p*K*i values of the less active molecules from the training set of the cluster I (LSL60111, LSL60112), whose nitrogen atom of imidazole is substituted with different alkyl groups. Based on these findings, we can conclude that substitution of imidazole nitrogen atoms negatively contributes to both I_2- IR and α_2- AR activity.

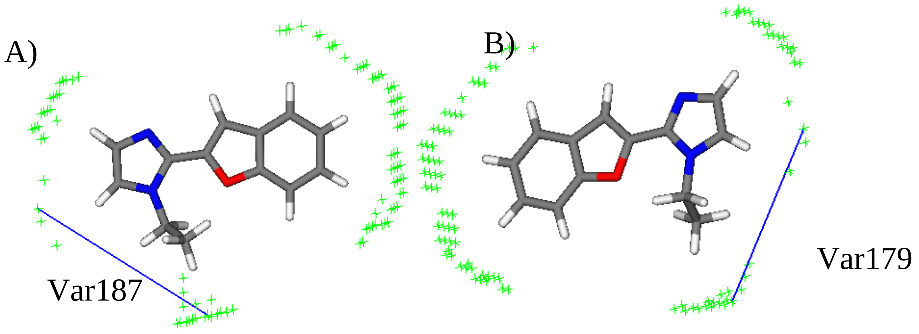

Figure S5. Unfavourable Var187: TIP-TIP (5A-3D-QSAR (I_2-IR) model**)** and unfavourable Var179: TIP-TIP (5B-3D-QSAR (α_2-AR) model) of compound LSL60112; the steric hot spots (TIP) are presented in green.

The introduction of a hydroxy or methoxy group on the carbon atom of the benzofuran correlates negatively with the activity of the cluster I compounds, as described by the unfavourable var 205 (TIP - TIP: 14.8Å - 15.2Å) and var195 (TIP - TIP: 10.8Å - 11.2Å). This is also confirmed by the additional unfavourable var 541 (N1 - TIP: 14.8Å - 15.2Å) observed between the steric hot spot around the imidazole and the hydrogen bond accepting group of the benzofuran (LSL60128, LSL60131, LSL60133) (Figure S6A). In contrast, var513 (N1 - TIP: 10.8Å - 11.2Å) and var457 (O – TIP: 10Å - 10.4Å) from the 3D-QSAR (α_2-AR) model show that the introduction of a hydrogen accepting group into the benzofuran increase the affinity of the molecules for α_2-AR (Figure S6B). Overall, we can conclude that the derivatives with the unsubstituted benzofuran moiety would have better I_2- IR activity and selectivity, which is consistent with the experimental results.

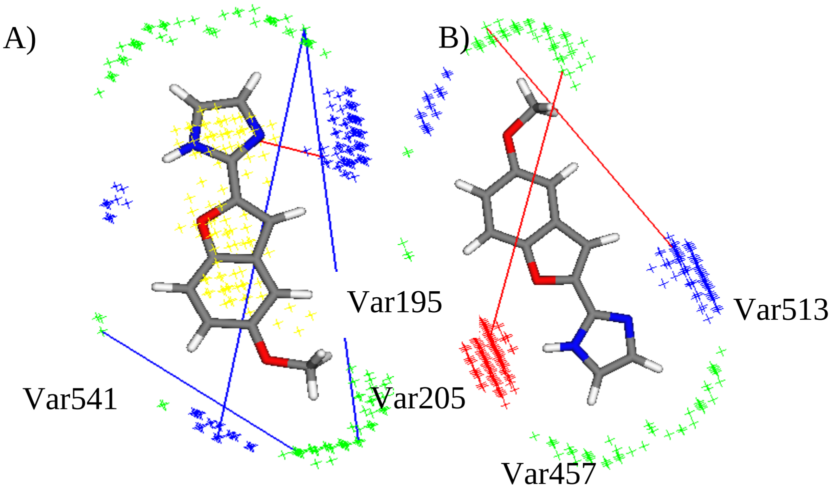

Figure S6. Unfavourable Var195, Var205: TIP-TIP and Var541: N1-TIP (6A-3D-QSAR (I_2-IR) model**)** and favourable Var513: N1-TIP and Var457: O-TIP (6B-3D-QSAR (α_2-AR) model) of compound LSL60131; the steric hot spots (TIP) are presented in green, H-bond donor regions in red and H-bond acceptor regions in blue.

For cluster II, the favourable var 285 underline the distance between the hydrogen donating group (oxygen atom of the carbonyl of maleimide) and the hydrophobic spot around the phenyl ring on the nitrogen atom of maleimide. This variable is not described for the compounds with alkyl groups on the nitrogen atom (B51 and B54). Moreover, the unfavourable var510 (N1- TIP: 2.4Å - 2.8Å) is present only in these two compounds of the cluster II and is observed between the oxygen atom of the carbonyl of maleimide and the alkyl groups on the nitrogen atoms. In view of these results, we can assume that this part of the molecule can be modified in order to increase the I_2- IR activity (Figure S7).

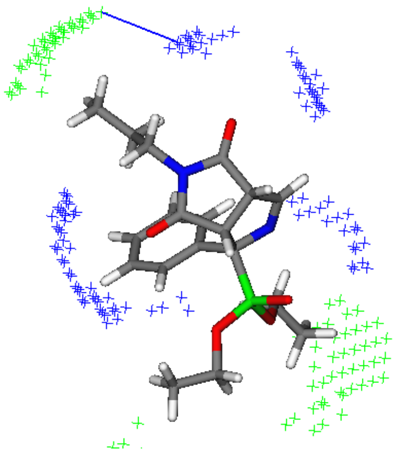

Figure S7. Unfavourable Var510: N1- TIP of compound **B51** (3D-QSAR (I_2-IR) model), the steric hot spots (TIP) are presented in green and H-bond acceptor regions in blue.

Introduction the substituents in the p-position of the phenyl ring (on the nitrogen atom of the maleimide) contribute negatively to the affinity of the molecules for the binding site of the I_2-IR receptor (B22, B62, B64) (Figure S8A). Also, the introduction of bulky substituents on the nitrogen atom of the maleimide is related to the decrease in the I_2-IR activity (B33, B34) (Figure S8B). This is explained by the unfavourable variables 205 (TIP - TIP: 14.8Å - 15.2Å). Therefore, we hypothesized that modification of this part of the molecule with small substituents in *meta*-position (such as a methyl group) will lead to an increase in the I_2-IR.

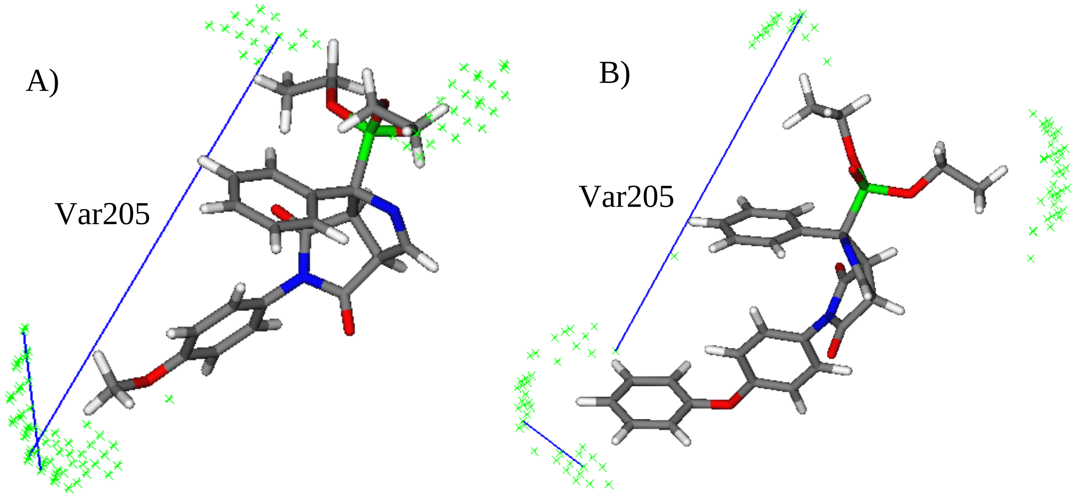

Figure S8. Unfavourable Var205: TIP-TIP (3D-QSAR (I_2-IR) model) of compounds B22 (8A) and B34 (8B); the steric hot spots (TIP) are presented in green.

Analysis of the 3D-QSAR (α_2-AR) model also reveals the presence of var136 (N1 – N1: 11.2Å – 11.6Å) in the least active molecule CR4056 (clinical candidate), which is formed between the hydrogen-accepting group (unsubstituted nitrogen atom) of the imidazole ring and the hydrogen-accepting group of quinazoline. This variable strongly decreases the α_2-AR activity, whereas it is not observed in the 3D-QSAR model for I_2 (Figure S9A). Moreover, unfavourable var205 (TIP - TIP: 14.8Å - 15.2 Å; 3D-QSAR model for I_2) shows the negative effects of the distance between the hot spot around the imidazole and the hot spot around the phenyl ring (position C-2 of quinazoline) on I_2- IR activity (Figure S9B).

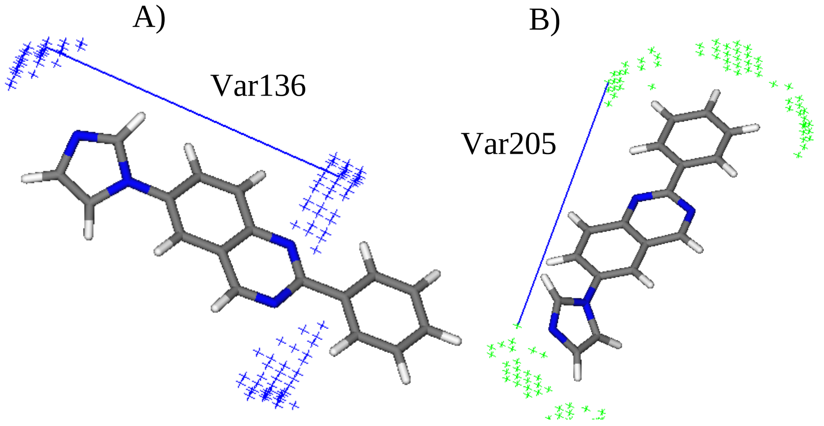

Figure S9. Unfavourable Var136: N1-N1 (9A-3D-QSAR (α_2-AR) model) and unfavourable Var205: TIP-TIP (9B-3D-QSAR (I_2-IR) model) of compound CR4056; the steric hot spots (TIP) are presented in green and H-bond acceptor regions in blue.

Based on the results and detailed analysis of the developed 3D QSAR models, we assume that:

1. Replacement of the benzofuran with a heterocycle such as quinazoline may be associated with an increase in I_2 selectivity in the I cluster, as variable 136 (N1-N1) has a negative effect on α_2-activity;
2. Changing the structure of CR4056 by removing the phenyl ring can increase I_2 activity by removing the negative impact of var205;
3. The use of unsubstituted imidazoline is crucial for I_2 activity, as demonstrated by the positive effect of var408, var179 and var187 (Cluster I);
4. Replacing the hydrogen-accepting group with electron-withdrawing groups can help increase I_2 activity by removing the negative effects of var195, var205, and var541. At the same time, this structural change may reduce the effects of var457

and var511, which are important for α_2-AR activity, which could correlate with the selectivity of the molecules (Cluster I)

5. The *p*-substitution of the benzene ring (link to the nitrogen atom of the maleimide) decreases the I_2 activity, so the structure of the compounds can be further modified in the *o*- or *m*-position in order to reduce the negative influence of var178 and var205 on the I_2 activity (Cluster II).

Compared to the results of the previous 3D-QSAR study for I_2-IR and α_2-AR, these new models have provided us with new conclusions that can be used in the design and synthesis of novel ligands. The main new findings we obtained from the detailed analyses of the new 3D-QSAR models are:

1) Replacement of benzofuran with quinazoline (with imidazoline at position C-6) is likely to increase activity and selectivity for I_2-IR due to the negative influence of var136 on α_2-activity;
2) Modification of bycyclic α-iminophosphonate derivatives such as the introduction of non-bulky substituents at the *meta*-position of the phenyl ring (on the nitrogen atom of the maleimide) and the removal of substituents at the *para*-position can increase the I_2- IR activity.
3) As with clinical candidate CR4056, removal of the phenyl group from the C-3 position of quinazoline could increase I_2- IR activity.

The applicability domain (AD)

Each 3D-QSAR model has its own applicability domain inside which the prediction activity is reliable. The leverage approach is used to define the applicability domain of 3D-QSAR models by creating a William plot. SPSS v.18.0 software was used for this procedure.[5] The critical leverage h* (the vertical line) is calculated according to the following equation (Eq. 8):

$$\mathbf{h^* = 3(p+1)/n} \quad (8)$$

[5] SPSS Inc. Released 2009. PASW Statistics for Windows, Version 18.0. Chicago: SPSS Inc.

where n represents the number of compounds in the training set, while p represents GRIND variables used to define AD. If the absolute standardized residual for a molecule exceeds three standard deviation units and/or the leverage value of a compound is greater than the critical value (h*), the compound is outside the AD of the model and the models generated are not reliable for predicting the activity of new compounds. **Figure S10**. shows that all compounds are in chemical space defined by AD for both 3D-QSAR models.

A)

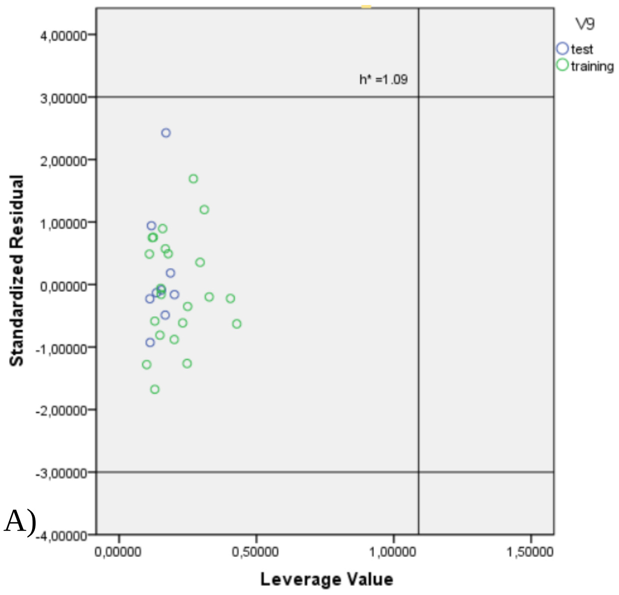

B)

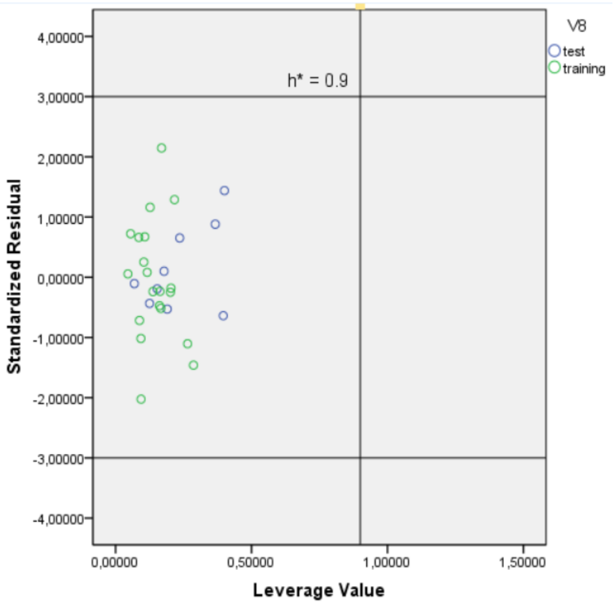

Figure S10. Applicability domain of developed 3D-QSAR (I_2-IR) model (10A) and 3D-QSAR (α_2–AR) model (10B).

Animals for PK studies

The pharmacokinetic study was carried on in male CD1 mice (Envigo Laboratories) with a body weight between 40 to 50 g (n = 3-4 per group). Animals were randomized to be included in the treated or control groups. A single intraperitoneal dose of LSL33 (2 mg/kg, 10 ml/kg) was administered early in the morning (between 8 and 11 a.m.) without anaesthesia. Compound was dissolved in 10 % of 2-hydroxypropyl)-β-cyclodextrin in physiological saline). Mice were monitored for signs of pain or distress during the time between injection and euthanasia. Mice were sacrificed by cervical dislocation and blood (0.6 mL) was collected at different time points (0 min, 5 min, 10 min, 15 min, 30 min, 45 min, 60 min, 2 h, 3 h, 4 h, 6 h, 8 h and 24 h after injection) in tubes with serum gel and clotting activator (Sarstedt Micro tube 1.1 mL Z-Gel). Samples were centrifugated at 10.000 rpm for 10 min to obtain plasma and stored at -80 ºC up to analysis of compound concentration by UPLC-MS/MS. Experimental procedures were in line with the Directive 2010/63/EU and approved by the Institutional Animal Care and Generalitat de Catalunya (#10291, 1/28/2018).

Metabolic stability of LSL33 in human liver microsomes

The reaction was initiated by adding HLM (1 mg/mL) to an equal volume of buffer solution containing the test compound and cofactors at the proper concentrations. Reactions without NADPH were also incubated to rule out non-NADPH metabolism or chemical instability in the incubation buffer. One positive control compound (verapamil) was included to monitor incubation course. All reactions were terminated using 60 µL of ice-cold acetonitrile at 0, 5, 15, 30, 45, and 60 min. The plates were centrifugated at 3500 rpm for 15 min. All experiments were conducted in triplicate. Samples were monitored to parent compound disappearance by LC-MS in MRM mode according to a quantitation method for each compound.
LSL33 was dissolved in DMSO at 0.5 mg/mL. Assay conditions, total incubation volume 400 µL, protein concentration 1 mg/mL, NADPH, 1.3 mM. Preparation of reagents: Test compound, the final concentration in the assay. NADPH solution, a stock solution of 2.66 mM NADPH was prepared by dissolving appropriate amount of NADPH in 100 mM potassium phosphate buffer. Human liver microsomes and verapamil were purchased from BD and NADPH were purchased from Sigma-Aldrich, Germany. The equipment needed for the study was Beckman Coulter Refrigerated centrifuge, Agilent 1290 Infinity Liquid Chromatograph, CTC Analytics PAL HT-xt injector, Api4000 Mass Spectrometer.

Bio-Analysis: Samples were monitored for parent compound disappearance by LC-MS in MRM mode according to a previously verified quantitation method for each compound. Key instrumental and analytical conditions are shown in the Table S6.

Data Analysis: The peak area of analyte was used to calculate the percentage of remaining compound at each incubation time (Table S7). The natural logarithm of the percentage of reamining **LSL33** was plotted versus incubation time to calculate the half-life using the following equation: Half-life ($T_{1/2}$) (min) = 0.693/*k* (the slope of the natural log of the percent remaining *versus* time) see Table S8 and Figure S11 . Intrinsic clearance was determined by the following equation: $CL_{int\ H}$ = Ln 2/$t_{1/2}$ (min) x volume incubation (mL)/microsomal protein (mg) x 45 (MPPGL) 1500 g human liver/ 70 Kg human body weight. Units for CL_{int} are usually expressed as mL/min/mg microsomal protein (table S8), MPPGL = referred to as mg microsomal protein per gram liver. Verapamil was used as positive control (see Table S8 and Figure S12). The categories classification for intrinsic clearance are shown in Table S9.

***Table S6**. Summary of LC-MS conditions*

HPLC	Agilent 1290
MS/MS	Api4000 (SCIEX)
Software	Analyst
Ionization Mode	Electrospray positive
Sample matrix	Microsomes+ NADPH + buffer
Column	Discovery HS C18, 2.1 X 5 mm, 5µm
Mobile phase	[Water: Acetonitrile :0.1% Formic Acid]
Flow rate	0.4 mL/min
Source Temperature (ºC)	500
Injection Volume (µL)	5
Run time (min)	5

__Table S7__. Percentage of LSL33 remaining at each incubation time

LSL33	
+ NADPH	
Time (min)	% Compound remaining
0<br>5<br>15<br>30<br>45<br>60	100.0±9.0<br>4.6±0.4<br>0.1±0.1<br>0.2±0.1<br>1.3±0.2<br>0.2±0.3
Minus NADPH	
Time (min)	% Compound remaining
0<br>60	100.0±10.2<br>96.6±9.0

__Table S8__. Metabolic stability of LSL33 and verapamil in human liver microsomes

Compound	Intrinsic clearance (mL/min/mg protein)	t1/2 (min)
LSL33	422	1.58
Verapamil	125	5.35

__Table S9__. Compound clearance category classification

Clearance Category	Intrinsic Clearance (mL/min/mg protein)
	Human Liver microsomes
Low	< 8.6
High	> 47.0

__Figure S11__. Time course of metabolic stability of LSL33 in human liver microsomes

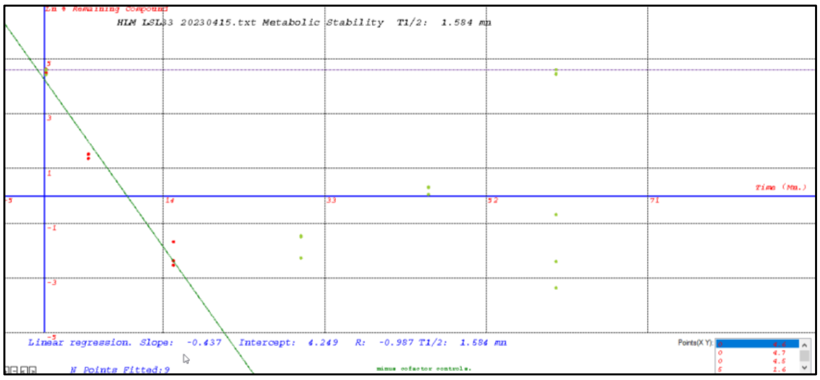

***Figure S12**. Time course of metabolic stability of verapamil in human liver microsomes*

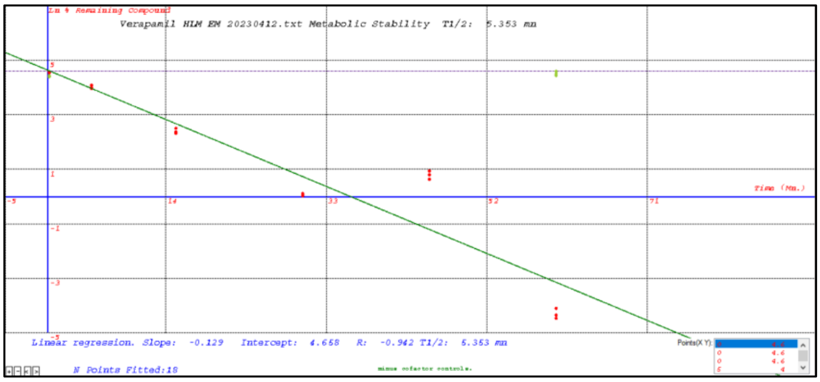

Method validation for quantification of LSL33 in Mouse Plasma

The objective of the study was to develop and validate a quantization method for LSL33 in mouse plasma using liquid chromatography coupled to mass spectrometry (LC-MS).

The internal standard used was LSL60101 (Figure S1).

Instrumentation: Mass spectrometer: Api4000 SCIEX; HPLC system: Agilent 1290, Analytical HPLC column: C18 2.1 x 5 mm, 3 µm (Supelco). Column oven: Agilent 1290. Auto sampler: CTC Pal-xt. Mobile Phase A: Water/Acetonitrile (90/10), 0.1% Formic acid. Mobile Phase B: Acetonitrile/Water (90/10), 0.1% Formic acid. Rising solution A: Water. Rising solution B: Acetonitrile/Water (80/20). Chromatographic conditions: Chromatographic column: C18 2.1x 5 mm, 3 µm (Supelco). Flow rate: 0.4 mL. Oven temperature: 20 ºC. Injection volume: 5 µL. Run time: 5.0 min. Auto-sampler temperature: 4 ºC. Gradient.

Mass spectrometric conditions: Detection of analyte and internal standards were carried out in triple quadrupole mass spectrometry with electrospray positive ionization. Mass/charge used in the present method are summarized below. m/z analyte: 201.088/160.206; 201.088/89.047; m/z internal standard 185.059/157.053. Parameters, voltage: 5000 V, temperature 500 ºC. Standard stock solutions: independent stock solutions of analyte an internal standard was prepared in 100% DMSO to get a concentration of 0.5 mg/mL. Calibration curve solutions were prepared and diluted with 100% DMSO, from the previous combined standard stock solution to achieve a concentration range from 4 ng/mL to 512 ng/mL for LSL33. Standard curve preparation. Aliquot volumes of the previous standard calibrations curve solutions were added to interference free plasma to prepare calibration curve, ranging from 4 ng/mL to 512 ng/mL for LSL33. Internal standard combined solution was transferred into a final volume of 10 mL acetonitrile to get a final concentration of 250 ng/mL.

Quality control samples (QC) were prepared from an independent analyte standard solution preparation (standard quality control solution). Aliquots volumes of the prepared standard quality control solution were dispensed into the interference free plasma to prepare the QC samples.

Standard Calibration Curve Samples, Standard Quality Control Samples and Standard Blank. Samples 1. Standard blank samples were prepared by dispensing 2 µL of 100% DMSO into 50 µL of Plasma. 2. Calibration Curve samples were prepared by dispensing 2 µL of standard calibration curve solution into 50 µL of Plasma. 3. Standard Quality control samples were prepared by dispensing 2 µL of standard QC solution into 50 µL of Plasma.

Chromatography: LSL33 displayed a retention time about 1.70 minutes. For LSL60101 the retention time was established at 1.56 minutes. Figures S13 and S14 display chromatogram for analyte and Internal standard for blank and lower limit of quantitation samples.

Specificity was investigated using six blank mouse plasma samples. No significant interferences were observed at the retention time of analyte and internal standards. The results are shown in Table S10 and Figure S13.

For LSL33 the lower and upper limits of quantification were established at 4 ng/ml and 512 ng/ml respectively.

Standard Calibration Curves: Two independent standard curves in mouse Plasma homogenate samples were prepared and analyzed covering the range of 4 and 512 ng/mL for LSL33. Calibration Curves linearity was higher than 0.995 over the range for LSL33. The peak area ratio of LSL 33 to the internal standard is related to concentration of internal standard using a linear fit, with 1/x (where x is concentration) weighting. Figure S15 is representative standard curve for LSL33 in mouse plasma samples. All standard Calibration curve samples back calculated concentrations met the acceptance criteria for precision (CV ± 20.0 %) and accuracy (± 20.0 % of theoretical concentration). Standard Calibration curve samples calculated concentrations in mouse plasma samples are displayed in Tables S11.

Method precision and accuracy were determined using 6 replicates of QC samples in mouse Plasma samples at three concentration levels. All QC samples met the acceptance criteria for Precision (CV ± 20.0 %) and Accuracy (± 20.0 % of theoretical concentration) for LSL33.

The method results for precision (CV %) and accuracy (%) of the QC samples of LSL 33 in mouse plasma samples are presented in Tables S12.

Method recovery of LSL33 was investigated at three concentration levels (10, 100, 250 ng/mL).

Analyte peak area at the established retention time in mouse Plasma homogenate QC samples was compared to analyte peak area at the established retention time in non-matrix based samples in order to calculate the method recovery.

Mean recovery values for LSL33 are 100, 82.7 and 84.7% at 10, 100, 250 ng/ml concentration levels, respectively.

The carry-over was checked for analyte and internal standard, the results are shown in Table S13.

***Figure S13**. Double Blank Sample Chromatogram of LSL33 and Internal Standard in mouse plasma samples*

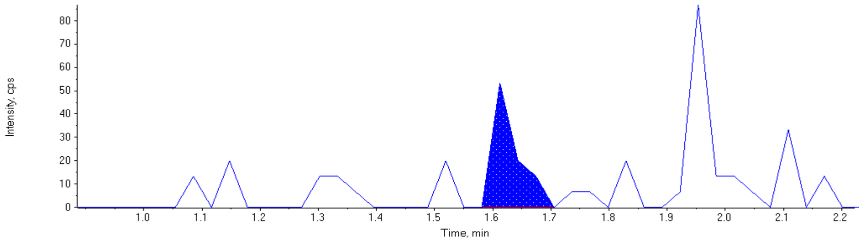

A. Peak of LSL33

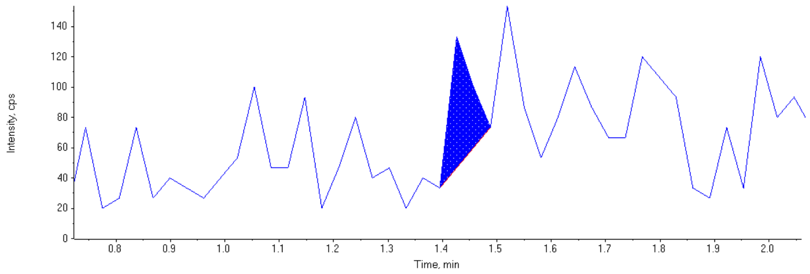

B. Peak of Internal standard

Figure S14. *Chromatogram of LSL33 and internal standard in LLOQ sample in mouse plasma samples (Concentration 4 ng/mL)*

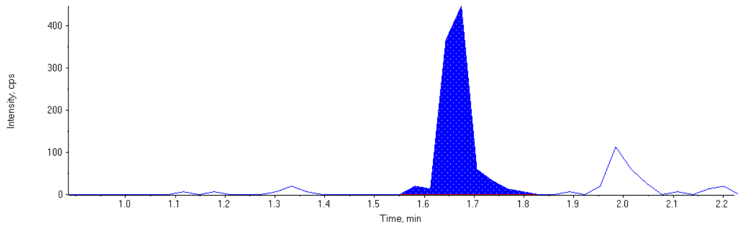

A. Peak of LSL 33

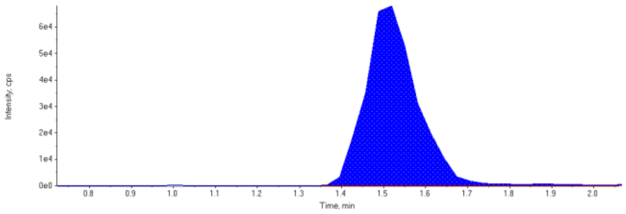

B. Peak of internal standard

Table S10*. Specificity of LSL33 and internal standard in mouse plasma samples*

	Peak area at LSL33 RT in Blank	Peak area at LSL33 RT in LLOQ Sample	Blank Peak area at LSL33 RT (% of LLOQ)	Peak area at IS RT in Blank	Peak area at IS RT in LLOQ Sample	Blank Peak area at IS (% of LLOQ)
Blank 1	1280	5300.0	24.1	236.0	583000.0	0.04
Blank 2	335.0	4790.0	6.9	260.0	629000.0	0.04
Blank 3	446.0	5220.0	8.5	397.0	603000.0	0.07
Blank 4	298.0	5270.0	5.6	260.0	593000.0	0.04
Blank 5	533.0	5180.0	10.3	223.0	568000.0	0.04
Blank 6	322.0	4950.0	6.5	298.0	606000.0	0.05
n	6	6	6	6	6	6
Mean	564.0	4123.3	10.3	279.0	597000.0	0.05

Figure S15*. Standard calibration curve of LSL33 in mouse plasma samples*

Regression Equation: y = 0.00163 x + 0.00324 (r = 0.9972) (weighting: 1 / x)

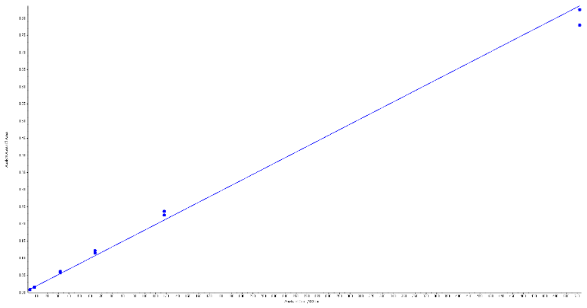

Table S11*. Back calculated values (ng/mL) data of calibration curve standard for LSL33 in mouse plasma samples*

STD Conc. (ng/ml)	4	8	32	64	128	512
Run 1	3.6	7.6	35.5	73.2	136.8	477.2
Run 2	3	7.4	33.6	69	143.7	505.4
Mean Calculated Concentration (ng/mL)	3.3	7.5	34.6	71.1	140.1	491.3
Accuracy (%)	82.1	93.3	100.8	111.1	109.6	96
Precision (CV %)	11.9	1.9	3.8	4.2	3.5	4.1
n	2	2	2	2	2	2

Table S12*. Accuracy and Precision of LSL33 in mouse plasma samples*

**Quality Control**	**LQC (4)**	**MQC (171)**	**HQC (512)**
**Concentration (ng/ml)**	2.9	200.8	529.1
	3.3	184.3	458.5
	3.5	192.0	481.5
	3.6	203.7	483.0
	3.0	200.4	487.6
		183.9	
**Mean**	3.3	194.2	488.0
**Precision (CV %)**	9.6	4.5	5.3
**Accuracy (%)**	81.5	113.6	95.3
n	5	6	5

__Table S13__. Carry over test of LSL 33 and internal standard in mouse plasma samples

Sample Name	Analyte	IS	% Carry Over	
			Analyte	IS
STD BLANK	384	1330		
LLOQ	5430	755000		
ULOQ	573000	845000		
STD BLANK	470	995	8.7	0.2

Method validation for quantification of LSL33 in Mouse Brain

The objective of the study was to develop and validate a quantization method for LSL33 in mouse plasma using liquid chromatography coupled to mass spectrometry (LC-MS).

The internal standard used was LSL60101 (Figure S1).

The instrumentation, materials and chromatographic conditions, and other general parameters are the same as reported in the method validation for quantification of LSL33 in mouse plasma.

Chromatography: LSL33 displayed a retention time about 1.99 minutes. For LSL60101 the retention time was established at 1.89 minutes. Figure S16 and S17 display chromatogram for analyte and Internal standard for blank and lower limit of quantitation samples.

Specificity was investigated using six blank mouse brain samples. No significant interferences were observed at the retention time of analyte and internal standards (see Table S14).

For LSL33 the lower and upper limits of quantification were established at 8 ng/ml and 512 ng/ml respectively.

Standard Calibration Curves: Two independent standard curves in mouse brain homogenate samples were prepared and analyzed covering the range of 8 and 512 ng/mL for LSL33. Calibration Curves linearity was higher than 0.995 over the range for LSL33. The peak area ratio of LSL33 to the internal standard is related to concentration of internal standard using a linear fit, with 1/x (where x is concentration) weighting. Figure S18 is representative of the standard curve for LSL33 in mouse brain samples. All standard Calibration curve samples back calculated concentrations met the acceptance criteria for precision (CV ± 20.0 %) and accuracy (± 20.0 % of theoretical concentration).

Standard Calibration curve samples back calculated concentrations in mouse brain homogenate samples are displayed in Table S15.

Method precision and accuracy were determined using 6 replicates of QC samples in mouse brain samples at two concentration levels. All QC samples met the acceptance criteria for Precision (CV ± 20.0 %) and Accuracy (± 20.0 % of theoretical concentration) for LSL33.

The method results for precision (CV %) and accuracy (%) of the QC samples of LSL33 in mouse Brain homogenate samples are presented in Table S16.

Method recovery of LSL33 was investigated at three concentration levels (10, 100, 250 ng/mL).

Analyte peak area at the established retention time in mouse brain homogenate QC samples as compared to analyte peak area at the established retention time in non-matrix based samples in order to calculate the method recovery.

Mean recovery values for LSL33 are 100, 83 and 100% at 10, 100, 250 ng/ml concentration levels, respectively.

The carry-over was checked for analyte and internal standard, the results are shown in Table S17.

Figure S16*. Double blank sample chromatogram of LSL33 and internal standard in mouse brain samples*

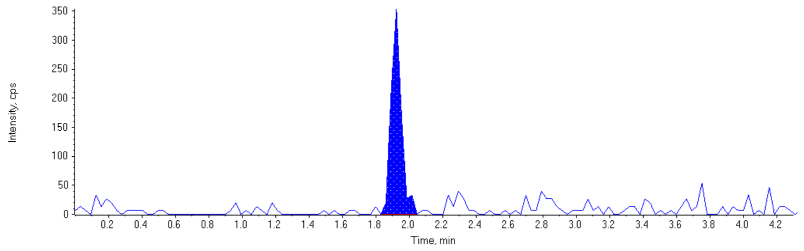

A. Peak of LSL33

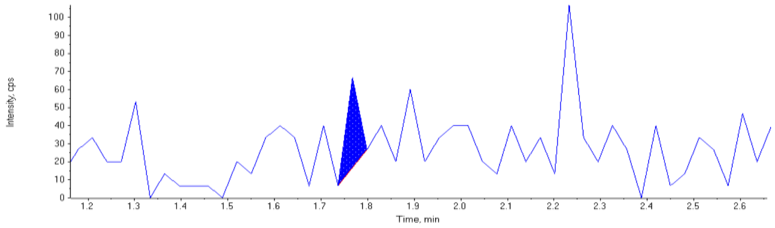

B. Peak of Internal standard

Figure S17. Chromatogram of LSL33 and internal standard in LLOQ sample in mouse brain samples (Concentration 8 ng/mL)

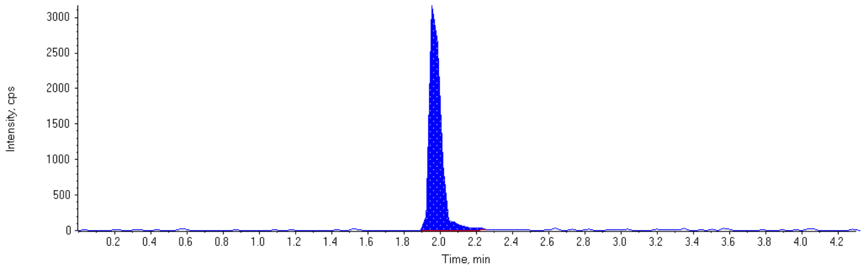

A. Peak of LSL 33

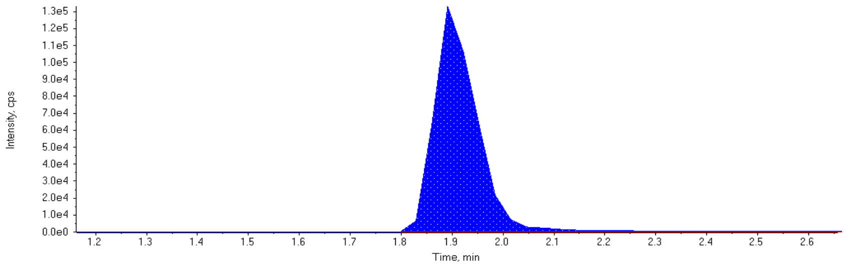

Peak of internal standard

Figure S18*. Standard curve of LSL33 in mouse brain samples*

Regression Equation: y = 0.00162 x + 0.00384 (r = 0.9951) (weighting: 1 / x)

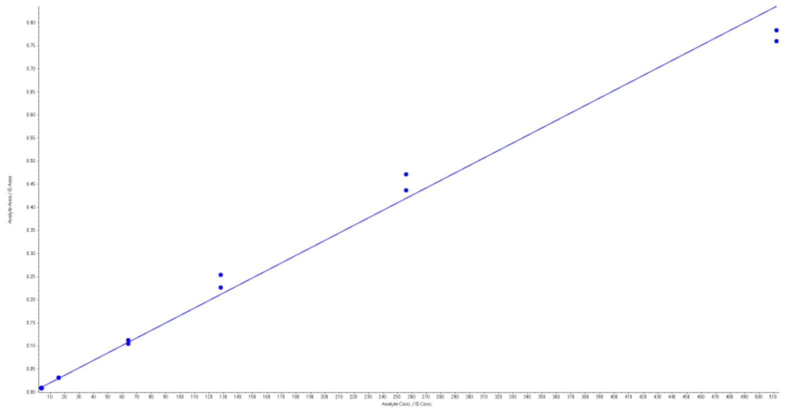

Table S14*. Specificity of LSL33 and internal standard in mouse brain samples*

	Peak area at LSL33 RT in Blank	Peak area at LSL33 RT in LLOQ Sample	Blank Peak area at LSL33 RT (% of LLOQ)	Peak area at IS RT in Blank	Peak area at IS RT in LLOQ Sample	Blank Peak area at IS (% of LLOQ)
Blank 1	1510	13800	10.9	533.0	1530000.0	0.04
Blank 2	2110	13600	15.5	620.0	1460000.0	0.04
Blank 3	2160	16600	13.0	558.0	1470000.0	0.04
Blank 4	1710	17300	9.9	422.0	1510000.0	0.03
Blank 5	1930	17200	11.2	273.0	1610000.0	0.02
Blank 6	1770	16600	10.7	341.0	1360000.0	0.02
n	6	6	6	6	6	6
Mean	1865.0	15850	11.9	457.8	1490000.0	0.03

Table S15*. Back calculated values (ng/mL) data of calibration curve standard for LSL33 in mouse Brain samples*

Standard Concentration (ng/ml)	8	16	64	128	256	512
Run 1	7.3	15.3	60.9	153.8	267.3	467.4
Run 2	7.1	15.4	65.8	136.8	288.6	482.2
Mean Calculated Concentration (ng/mL)	7.2	15.3	63.4	145.3	277.9	474.8
Accuracy (%)	90.2	95.9	99.0	113.5	108.6	92.7
Precision (CV %)	1.5	0.6	5.3	8.3	5.4	2.2
n	2	2	2	2	2	2

Table S16. *Accuracy and precision of LSL33 in mouse brain samples*

Quality Control	MQC (57)	HQC (512)
Concentration (ng/ml)	68.7	493.9
	63.4	499.2
	72.4	527.9
	60.4	503.5
	67.0	570.8
	60.2	497.2
Mean	65.3	515.4
Precision (CV %)	7.5	5.8
Accuracy (%)	114.6	100.7

Table S17. *Carry over test of LSL 33 and internal standard in mouse brain samples*

Sample Name	Analyte	IS	% Carry Over	
			Analyte	IS
STD BLANK	1120	149		
LLOQ	16500	1500000		
ULOQ	1040000	1440000		
STD BLANK	976	805	6.8	0.05

Table S18. Antibodies used in Western blot studies.

Antibody	Host	Source/Catalog	WB dilution
**Tubulin**	Mouse	Anti-a-Tubulin/DM1A	1:2000
**PSD95**	Rabbit	Gene Tex/133091	1:1000
**Goat-anti-mouse HRP conjugated**		BioRad/170-5047	1:2000
**Goat-anti-rabbit HRP conjugated**		BioRad/170-6515	1:2000

Table S19. Syber Green Primers used in qPCR studies.

TARGET	FORWARD PRIMER (5'-3')	REVERSE PRIMER (5'-3')
Bdnf	TGCGAGTATTACCTCCGCCAT	TCACGTGCTCAAAAGTGTCAG
Ngf	GGAGCGCATCGAGTGACTT	CCTCACTGCGGCCAGTATAG
Tnf-α	TCGGGGTGATCGGTCCCCAA	TGGTTTGCTACGACGTGGGCT
Il-6	ATCCAGTTGCCTTCTTGGGACTGA	TAAGCCTCCGACTTGTGAAGTGGT
β-Actin	CAACGAGCGGTTCCGAT	GCCACAGGTTCCATACCCA
